# The degradation of performance of a state-of-the-art skin image classifier when applied to patient-driven internet search

**DOI:** 10.1038/s41598-022-20632-7

**Published:** 2022-09-28

**Authors:** Seung Seog Han, Cristian Navarrete-Dechent, Konstantinos Liopyris, Myoung Shin Kim, Gyeong Hun Park, Sang Seok Woo, Juhyun Park, Jung Won Shin, Bo Ri Kim, Min Jae Kim, Francisca Donoso, Francisco Villanueva, Cristian Ramirez, Sung Eun Chang, Allan Halpern, Seong Hwan Kim, Jung-Im Na

**Affiliations:** 1 Department of Dermatology, I Dermatology Clinic, Seoul, Korea; 2 IDerma Inc., Seoul, Korea; 3 Department of Dermatology, School of Medicine, Pontificia Universidad Católica de Chile, Santiago, Chile; 4 Department of Dermatology, University of Athens, Andreas Syggros Hospital of Skin and Venereal Diseases, Athens, Greece; 5 Department of Dermatology, Sanggye Paik Hospital, Inje University College of Medicine, Seoul, Korea; 6 Department of Dermatology, Dongtan Sacred Heart Hospital, Hallym University College of Medicine, Seoul, Korea; 7 Department of Plastic and Reconstructive Surgery, Kangnam Sacred Heart Hospital, Hallym University College of Medicine, 1, Singil-ro, Yeong deong op-gu, Seoul, 07441 Korea; 8 Department of Dermatology, Seoul National University Bundang Hospital, 82 Gumi-Ro 173 Beon-Gil, Seongnam, 463-707 Gyeonggi Korea; 9 Department of Dermatology, Asan Medical Center, Ulsan University College of Medicine, Seoul, Korea; 10 Dermatology Service, Department of Medicine, Memorial Sloan Kettering Cancer Center, New York, NY USA

**Keywords:** Machine learning, Skin diseases, Skin cancer

## Abstract

Model Dermatology (https://modelderm.com; Build2021) is a publicly testable neural network that can classify 184 skin disorders. We aimed to investigate whether our algorithm can classify clinical images of an Internet community along with tertiary care center datasets. Consecutive images from an Internet skin cancer community (‘RD’ dataset, 1,282 images posted between 25 January 2020 to 30 July 2021; https://reddit.com/r/melanoma) were analyzed retrospectively, along with hospital datasets (Edinburgh dataset, 1,300 images; SNU dataset, 2,101 images; TeleDerm dataset, 340 consecutive images). The algorithm’s performance was equivalent to that of dermatologists in the curated clinical datasets (Edinburgh and SNU datasets). However, its performance deteriorated in the RD and TeleDerm datasets because of insufficient image quality and the presence of out-of-distribution disorders, respectively. For the RD dataset, the algorithm’s Top-1/3 accuracy (39.2%/67.2%) and AUC (0.800) were equivalent to that of general physicians (36.8%/52.9%). It was more accurate than that of the laypersons using random Internet searches (19.2%/24.4%). The Top-1/3 accuracy was affected by inadequate image quality (adequate = 43.2%/71.3% versus inadequate = 32.9%/60.8%), whereas participant performance did not deteriorate (adequate = 35.8%/52.7% vs. inadequate = 38.4%/53.3%). In this report, the algorithm performance was significantly affected by the change of the intended settings, which implies that AI algorithms at dermatologist-level, in-distribution setting, may not be able to show the same level of performance in with out-of-distribution settings.

## Introduction

The advent of convolutional neural networks (CNNs) and the invaluable role of better quality and larger datasets have led to remarkable advances in both clinical images and dermoscopy machine learning algorithms^1–5^. For clinical image analysis, several studies have showed dermatologist-level performance in retrospective experimental settings. Fujisawa et al. developed a 14-disease classifier using 4,867 skin tumour images, with a Top-1 accuracy of 76·5%^6^. Liu et al. developed a 419-disease classifier using 64,837 images, and their system increased the Top-1 accuracy of primary care physicians from 48 to 58% in a reader test^7,8^. Masaya et al. reported on a 59-disease classifier developed using 70,196 images, with a Top-1 accuracy of 57.9%^9^.

However, most algorithms have been tested using a small number of internal datasets generated by specialists in tertiary care centres. Although the expertise of a user can affect an algorithm’s performance in real-world settings, few studies used images captured by patients; in contrast, most studies have been conducted using private datasets, and few benchmark studies have been performed^10,11^.

In medical research, very few prospective studies have been reported, and there are only 11 randomized controlled trials evaluating deep learning tools^12,13^. Considering that most of the failed prospective studies are not reported, it is doubtful whether the retrospective results of deep learning based algorithms are predictive of the real-world performance. In computer vision research, the change of object backgrounds and imaging viewpoints made a 40–45% drop in performance of the state-of-art CNNs^14^. This implies that the performance of a CNN is vulnerable to out-of-distribution setting and a rigorous validation with diverse datasets is required to check the generalizability of algorithms.

Model Dermatology (https://modelderm.com) is an ensemble of CNNs that can classify 184 skin diseases^15–17^. Because algorithm performance may vary depending on intended uses, we evaluated using various datasets with different characteristics. To validate the algorithm using layperson-captured images, we created a dataset comprising images posted by an Internet melanoma community (The ‘RD dataset’, Table 1). Additionally, we tested the algorithm using datasets obtained through teledermatological (TeleDerm dataset^18^) and conventional dermatological care (SNU dataset^17^ and Edinburgh dataset). In this study, the standalone performance in terms of Top accuracy in multi-class classification, sensitivity, specificity, and area under the curve (AUC) in binary classification for determining malignancy were analysed. The aim of this study is to demonstrate how the performance of algorithms is affected by the change in settings by comparing the performance of the algorithm on datasets with different characteristics. In particular, the performance of the algorithm using the community images was compared because the community images are the typically out-of-distribution.

**Table 1:** Summary of the test datasets.

	RD	SNU	Edinburgh	TeleDerm
Number of cases	1282	2101	1300	340
Source	Internet community	Tertiary care center	Tertiary care center	Teledermatology
Photographer	Patient	Physician	Professional photographer	Patient
Fitzpatrick skin type	–	3–4	1–2	1–4
Number of disease classes	62	133	10	87
**Disease category**				
Inflammatory Dermatitis	12 (0.9%)	131 (6.2%)	–	82 (24.1%)
Acne/rosacea	–	51 (2.4%)	–	78 (22.9%)
Autoimmune	2 (0.2%)	90 (4.3%)	–	34 (10.0%)
Papulosquamous	–	105 (5.0%)	–	17 (5.0%)
Others inflammatory	30 (2.3%)	159 (7.6%)	–	14 (4.1%)
Viral infection	39 (3.0%)	144 (6.9%)	–	22 (6.5%)
Fungal infection	7 (0.5%)	85 (4.0%)	–	20 (5.9%)
Bacterial infection	3 (0.2%)	125 (5.9%)	–	9 (2.6%)
Parasitic infection	–	15 (0.7%)	–	1 (0.3%)
Benign neoplastic	896 (69.9%)^a^	620 (29.5%)	819 (63.0%)	28 (8.2%)
Malignant neoplastic	123 (9.6%)^a^	182 (8.7%)	481 (37.0%)	4 (1.2%)
Alopecia, scarring	–	–	–	8 (2.4%)
Alopecia, non-scarring	–	20 (1.0%)	–	7 (2.1%)
Others	170 (13.3%)	374 (17.8%)	–	16 (4.7%)

^a^The ground truth of the RD dataset was voted on by five specialists, whereas the malignancies in the other datasets were determined by pathological examinations.

## Results

Top-(n) accuracy represents that the correct diagnosis is among the top n predictions output by the model.

### Binary classification of malignant versus benign disorders

**Figure 1:**
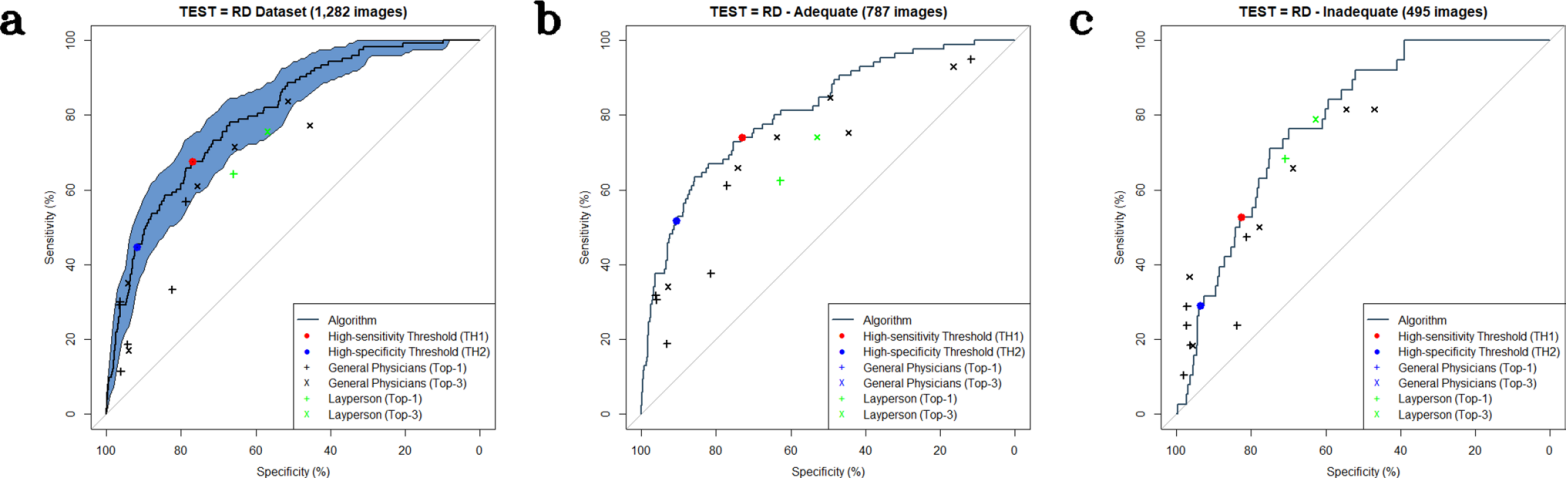
Binary classification for determining suspected malignancy using the Internet community (RD) dataset. (**a**) TEST = RD dataset (1,282 images). (**b**) TEST = RD_adequate_ subset dataset (787 adequate images). (**c**) TEST = RD_inadequate_ subset dataset (497 inadequate images), Red dot (TH1)-The algorithm at the high sensitivity threshold, Blue dot (TH2)-The algorithm at the high specificity threshold, Black dots-the six general physicians, Green dots-the layperson (cluster), × − Sensitivity and specificity derived from the Top-3 diagnoses of the participants, + − Sensitivity and specificity derived from the Top-1 diagnoses of the participants, The shaded area indicates the 95% confidence interval.

**Figure 2:**
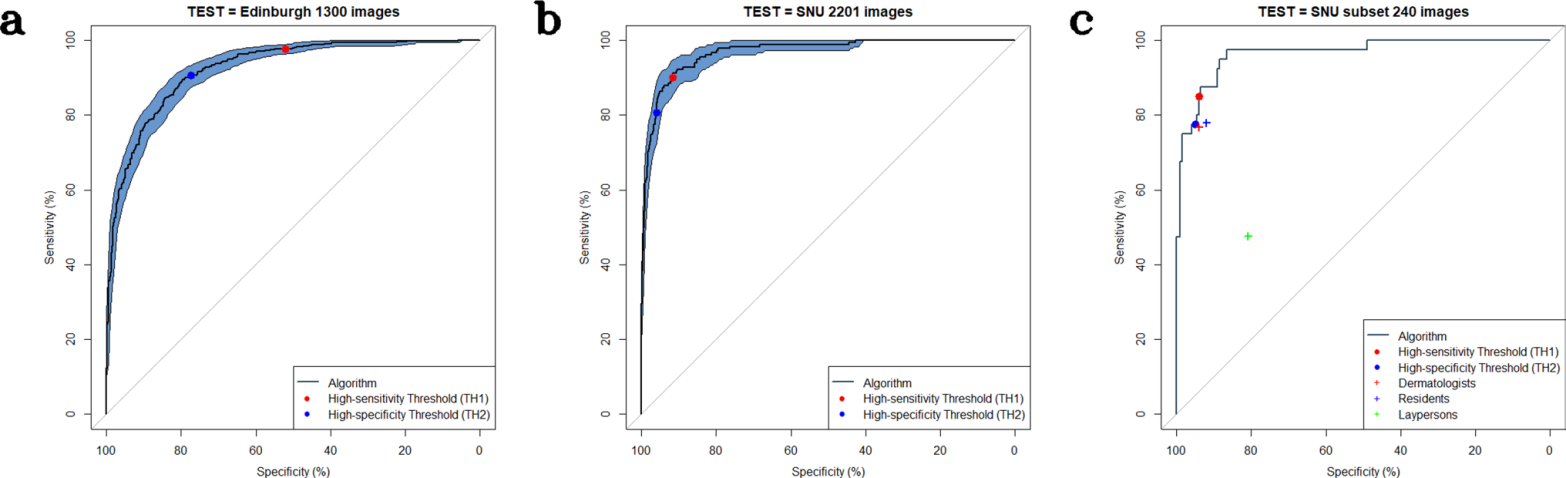
Binary classification for determining malignancy using the hospital (SNU and Edinburgh) datasets. (**a**) TEST = Edinburgh dataset (1,300 images). (**b**) TEST = SNU dataset (2,201 images). (**c**) TEST = SNU public subset dataset (240 images), Red dot (TH1)-The algorithm at the high sensitivity threshold, Blue dot (TH2)-The algorithm at the high-specificity threshold, + − Average of dermatologists, residents, and laypersons in the previous study^17^. The mean sensitivity/specificity using 240 test images was adapted, and the result of the reader study is available at https://doi.org/10.6084/m9.figshare.6454973, The shaded area indicates the 95% confidence interval. The TeleDerm dataset was excluded in this malignancy analysis because it includes only four malignancies.

When using the RD dataset, the AUC for determining malignancy was 0.800 (95% CI 0.761–0.839), which was lower than that obtained using the SNU dataset (0.969 CI 0.957–0.980; *P* < 0.0001, Delong method) and Edinburgh dataset (0.925 CI 0.910–0.939; *P* < 0.0001) (Figs. 1 and 2). In the subset analysis of the RD dataset, the AUC with the RD_adequate_ subset was 0.810 (CI 0.761–0.860), whereas that with the RD_inadequate_ subset was 0.784 (CI 0.722–0.845; *P* = 0.51 between RD_adequate_ and RD_inadequate_).

When using the *high sensitivity threshold*, sensitivity/specificity was 67.5 (CI 58.5–75.6)/77.0 (CI 74.4–79.3)% in the RD dataset, 90.1 (CI 85.1–94.0)/91.7 (CI 90.4–92.8)% in the SNU dataset, and 97.7 (CI 96.3–99.0)/52.0 (CI 48.5–55.6)% in the Edinburgh dataset. At the *high specificity threshold*, sensitivity/specificity was 44.7 (CI 36.6–53.7)/91.8 (CI 90.2–93.4)% in the RD dataset, 80.8 (CI 74.7–86.8)/95.9 (CI 95.0–96.8)% in the SNU dataset, and 90.6 (CI 87.9–93.1)/77.3 (CI 74.6–80.2)% in the Edinburgh dataset (Table 2). In the subset analysis of the RD dataset, sensitivity/specificity at the *high sensitivity threshold* with the RD_adequate_ subset was 74.1 (CI 64.7–82.4)/73.1 (CI 69.9–76.4), whereas sensitivity/specificity with the RD_inadequate_ subset was 52.6 (CI 36.8–68.4)/82.7 (CI 79.4–86.2). Sensitivity/specificity at the *high specificity threshold* with the RD_adequate_ subset was 51.8 (CI 41.2–62.4)/90.6 (CI 88.3–92.7), whereas sensitivity/specificity with the RD_inadequate_ subset was 29.0 (CI 15.8–42.1)/93.7 (CI 91.5–95.8).

**Table 2:** Sensitivity, specificity, positive predictive value, and negative predictive value in the binary-class classification.

Test dataset	Sensitivity	Specificity	PPV	NPV
**Binary Classification at High Sensitivity Threshold**				
RD/1,282 images	67.5 (58.5–75.6)	77.0 (74.4–79.3)	23.6 (20.8–26.6)	95.7 (94.6–96.7)
RDadequate/787 images	74.1 (64.7–82.4)	73.1 (69.9–76.4)	25.0 (21.8–28.3)	95.9 (94.5–97.2)
RDinadequte/495 images	52.6 (36.8–68.4)	82.7 (79.4–86.2)	20.2 (14.3–26.3)	95.5 (93.9–97.0)
SNU/2,201 images	90.1 (85.1–94.0)	91.7 (90.4–92.8)	50.6 (46.9–54.5)	99.0 (98.5–99.4)
SNU subset/240 images	85.0 (75.0–95.0)	94.0 (90.5–97.0)	74.4 (64.0–85.7)	96.9 (94.9–99.0)
Edinburgh/1,300 images	97.7 (96.3–99.0)	52.0 (48.5–55.6)	54.5 (52.7–56.4)	97.5 (95.9–98.8)
**Binary Classification at High Specificity Threshold**				
RD/1,282 images	44.7 (36.6–53.7)	91.8 (90.2–93.4)	36.7 (30.7–43.5)	94.0 (93.2–94.9)
RDadequate/787 images	51.8 (41.2–62.4)	90.6 (88.3–92.7)	40.0 (32.7–47.8)	94.0 (92.7–95.2)
RDinadequte/495 images	29.0 (15.8–42.1)	93.7 (91.5–95.8)	27.3 (15.8–40.5)	94.0 (93.0–95.2)
SNU/2,201 images	80.8 (74.7–86.8)	95.9 (95.0–96.8)	65.5 (60.1–70.6)	98.1 (97.6–98.7)
SNU subset/240 images	77.5 (62.5–90.0)	95.0 (92.0–97.5)	76.2 (64.6–86.9)	95.5 (92.8–97.9)
Edinburgh/1,300 images	90.6 (87.9–93.1)	77.3 (74.6–80.2)	70.1 (67.7–72.9)	93.4 (91.6–95.0)

*PPV* positive predictive value, *NPV* negative predictive value.

In the reader test, the mean sensitivity/specificity of the 32 laypersons (cluster), calculated from their Top-1 and Top-3 judgments were 64.2%/66.0% and 75.6%/56.9%, respectively. The mean sensitivity/specificity of the six general physicians, calculated from their Top-1 and Top-3 judgments were 30.0%/90.8% and 57.6%/71.1%, respectively.

### Multi-class classification for diagnosing general skin disorders

The Top-1/3 accuracies were 39.2%/67.2% (RD), 54.5%/77.6% (SNU), 64.0%/84.1% (Edinburgh), and 46.5%/66.8% (TeleDerm) (Table S2 for RD, S3 for Edinburgh, S4 for SNU, and S5 for SNU subset). In the RD dataset, the algorithm’s Top-1 accuracy (39.2%) was comparable to that of the six general physicians (36.8%; one-sample t-test, *P* = 0.25), but its Top-3 accuracy (67.2%) was higher than that of the general physicians (52.9%; one-sample t-test, *P* = 0.014). In the RD dataset, the Top-1/3 accuracy of the laypersons was only 19.2%/24.4% although Internet searches were allowed. In the SNU dataset, the algorithm’s Top-1/3 accuracy was comparable to that of two dermatologists (54.2%/71.8%) that was conducted in a previous study^17^. However, in the TeleDerm dataset, the algorithm’s Top-1 accuracy (46.5%) was still lower than that of dermatologists (60.1%) that was conducted in a previous study^18^.

In the subset analysis of the RD dataset, human readers and the algorithm reacted differently to images of inadequate quality. The algorithm’s Top-1/3 accuracy in the RD_adequate_ subset (43.2%/71.3%) was higher than that in the RD_inadequate_ subset (32.9%/60.8%; chi-squared test; *P* = 0.0003/*P* = 0.0001; Table S6). Unlike the deterioration in the algorithm’s performance, there was minimal difference in the physicians’ performance between the RD_adequate_ and RD_inadequate_ datasets (mean Top-1/3 of six GPs = 35.8%/52.7% versus 38.4%/53.3%; Table S7).

## Discussion

In this study, we demonstrated that our algorithm showed an equivalent diagnostic performance to dermatologists in classifying hospital images taken from tertiary care centres. The algorithm was also able to classify and triage suspected lesions from community images with a diagnostic performance similar to that of general physicians.

The data flow of dermatological evaluation starts with the patient’s decision to go for a medical evaluation (i.e. detecting a lesion of unknown significance by the patient). However, current images and metadata in available datasets are mostly derived from cases involving biopsies performed many levels above the flow of data, specifically, from tertiary care centres (or cancer centres). This is actually at the end of most data flow and likely represents only a minor portion of the real-world data. From the time a suspected lesion is identified at home to the time a biopsy is performed at the clinic, numerous cases are dropped from the data flow without being documented or included in currents datasets. Case selection depends on various factors, ranging from patient knowledge, socioeconomic status, among many others. In several instances, selections are also made due to the indications for laboratory tests in clinics. Cases without indications for biopsy or laboratory tests are not usually documented in detail, or photographs are simply not captured at all. Whether to perform a follow-up or not, and even the number of visits, may be affected by the severity or characteristics of the disease. Therefore, if a model is trained only with datasets and metadata originating from tertiary care centres, several biases would inevitably be transferred to the model.

We designed the present study to avoid biases that may result from testing only photographs captured by dermatologists at tertiary centres and test the algorithm in this real-world scenario. The following four combinations are plausible situations, which can vary based on whether the layperson suspects skin cancer or whether the doctor diagnoses skin cancer. We verified all these combinations using various datasets captured by both physicians (Edinburgh, SNU) and laypersons (RD, TeleDerm).

- Layperson suspects & doctor diagnoses cancer-RD, TeleDerm, SNU, Edinburgh.
- Layperson suspects & doctor diagnoses benign condition-RD.
- Layperson neglects & doctor diagnoses cancer-SNU, Edinburgh, TeleDerm.
- Layperson neglects & doctor diagnoses benign condition-non-lesional crops (ISIC dataset in the Supplementary Result).

In order to improve the poor generalizability of the old model, we attempted to improve the quality of the data (data-centric approach) rather than the architecture of the model (software-centric approach)^20^. First, we annotated a huge number of image crops to represent the various test situations. To improve the diagnosis of minor lesions that cannot be classified as a specific condition, a region-based CNN was used to detect various shapes of both lesional and non-lesional areas and to generate the training dataset^16,21^. Second, training data were reinforced by crawling the Internet to collect and annotate images of common disorders (e.g., contact dermatitis) that have high prevalence rates in the intended use setting as well as to improve diagnostic accuracy in subjects of various ethnicities. Although the ground truth was somewhat inaccurate, the performance of the CNNs was robust^22^, and we demonstrated that the model that was trained using a huge generated dataset (49,567 images) and that was based on image findings exhibited better performance in the binary classification of onychomycosis than the model trained with a smaller number of images (3,741 images), with accurate ground truths^21^. Finally, after removing unnecessary classes (e.g., graft-versus-host-disease) and adding classes corresponding to common conditions (e.g., erosion), 184 disease classes were finally selected to train the new model. With these enhancements, our algorithm exhibited dermatologist-level performance on using tertiary care datasets in the experimental setting where a diagnosis had to be reached based only on images provided (54.5% vs. two dermatologists, 54.2%; SNU, Table S4).

However, the accuracy is still not comparable to a dermatologist in a real-world setting. First, the algorithm was trained using data with limited relevance. Images do not provide enough information to accurately diagnose certain skin disorders (e.g. palpation, side illumination, context). Dermatologists in real-world clinical practice are much more accurate than in the reader test^23^ and in-person examination was important even in a study using dermoscopic images^24^.

Second, algorithms cannot predict at all for the out-of-distribution classes for which the algorithm was not trained. In the TeleDerm dataset, 10.3% of images (21 classes, 35 images) belonged to the out-of-distribution classes, and in the RD dataset, 1.3% of images (5 classes, 17 images) were out-of-distribution. As a result, the accuracy using the TeleDerm datasets was 46.5%, which was still inferior to that of dermatologists (60.1%)^17,18^. Third, photograph quality depends on a user’s skill and affects algorithm performance. In this study, the Top-1/3 accuracy with the RD_inadequate_ subset (32.9%/60.8%) was lower than that with the RD_adequate_ subset (43.2%/71.3%), which implies that the inadequateness of image quality significantly reduced algorithm performance, as previously reported^18^. Conversely, this marked deterioration with image quality was not observed in the human participants (Table S7), which was also previously reported^18^. In addition, not only did the performance decreased, the prediction of the algorithm at the cut-off thresholds changed to be very specific as shown in Fig. 2b. To detect poor-quality photographs, we developed a fine image selector^16^. This is similar to the methods of Vodrahalli et al. who developed an automated image assessment pipeline capable of rejecting 50% of subpar quality images, while retaining 80% of good-quality images^25^.

Due to these limitations, we hypothesize that our algorithm could be used an ‘assistant’ rather than a standalone ‘diagnostic tool’ at its current stage of development. Algorithms can provide second opinions and information regarding potential diagnoses, thereby augmenting a user’s judgment^18^. If a physician’s intelligence could be harmonised with the abstract experience of neural networks, ‘augmented intelligence’ may be achieved because the diagnostic pattern differs between the algorithm and participants (Figs. S2 and S3). In our reader test, the sensitivity and specificity were highly variable among physicians, as the decisions of some physicians were too specific while those of others were sensitive in determining malignancy. The algorithm may help to give a balanced second opinion close to the mean.

Finally, the algorithm (Top-1/3 = 39.2%/67.2%) could provide more accurate information than random Internet searches, and the algorithm’s accuracy was equivalent to that of general physicians (Top-1/3 = 36.8%/52.9%). In that respect, we hypothesize that the algorithm could ‘replace’ the role of the conventional internet searches. Social networks have become an essential tool for disseminating healthcare information; however, shared dermatology-related content is sometimes imprecise and confusing^26^, despite attempts to improve disease-related content based on evidence medicine^27^. The Top-1/3 accuracy of laypersons in the RD dataset was only 19.2%/24.4% despite Internet use being allowed (Table S2). Although there is extensive information available on the Internet, it is not easy for laypersons to find clues regarding diagnoses. The Top-3 accuracy of the laypersons (24.4%) was only slightly higher than their Top-1 accuracy (19.2%), which implies that merely narrow scope of information was delivered by a random Internet search.

## Limitations

First, the ground truth of the RD dataset was not determined based on biopsy results, although the malignancies of other test datasets were confirmed by pathological examinations. This has been used in prior studies, as biopsies are not usually performed in the most common dermatological diseases^8,18^. It is ideal to collect all continuous data flow of “layperson’s evaluation”-“physician’s in-person evaluation”-“pathologic result” in subsequent studies. However, by including non-biopsied cases, we widened the inclusion criteria probably avoiding datasets inclusion bias. Second, the images in the RD dataset were mostly captured by young people. Further investigations are required to assess accessibility and efficacy in the elderly. Older people might be less used to digital photography and might capture lower quality images. There may be also a selection bias given persons who posted on Reddit are more interested in their skin and active in the Internet community. Third, it would be ideal if U.S. physicians were included in the reader test because Reddit is mainly a U.S. melanoma community. Nevertheless, the 1,282 images were evaluated by the 6 readers from different countries and different backgrounds. This is a unique strength of the present study as few studies have had participants individually read and diagnose such a large number of images for comparison^28^. Finally, the performance of the algorithm was not assessed in darker skin populations in detail. More efforts are required to acquire clinical data to accurately assess skin disorders in the black population^29^.

In conclusion, the algorithm proposed here was shown to be capable of classifying tertiary care-originated datasets comparable to that of dermatologists. The algorithm was also able to triage Internet community-acquired images with the same accuracy level as that of general physicians, which is expected to be useful in a teledermatology environment. These results were achieved by augmenting a large number of training images based on the data-centric approach to address issues raised in academic discussions^18,19,30–32^. We demonstrated the degradation of performance from curated clinical images (SNU and Edinburgh dataset), teledermatology images (Telederm dataset), and community images (RD dataset). Our result implies that algorithms should be validated in the intended use setting because algorithms may show uncertainty in diverse real-world situations^33^. We expect the algorithm to be used as an adjunct tool that could aid medical professionals in diagnosing general skin disorders and in triaging suspected malignancies. Further prospective studies are required to investigate whether the algorithm can improve clinical outcomes in subjects of various ethnicities.

## Materials and methods

This study was reported per the STARD-2015^34^ reporting guidelines for diagnostic accuracy studies. With the approval of the institutional review board (Kangnam Sacred Hospital Institutional Review Board, #2021-07-019), the informed consent was waived for this retrospective study. All experiments were performed in accordance with the relevant guidelines and regulations.

### Test datasets

**Figure 3:**
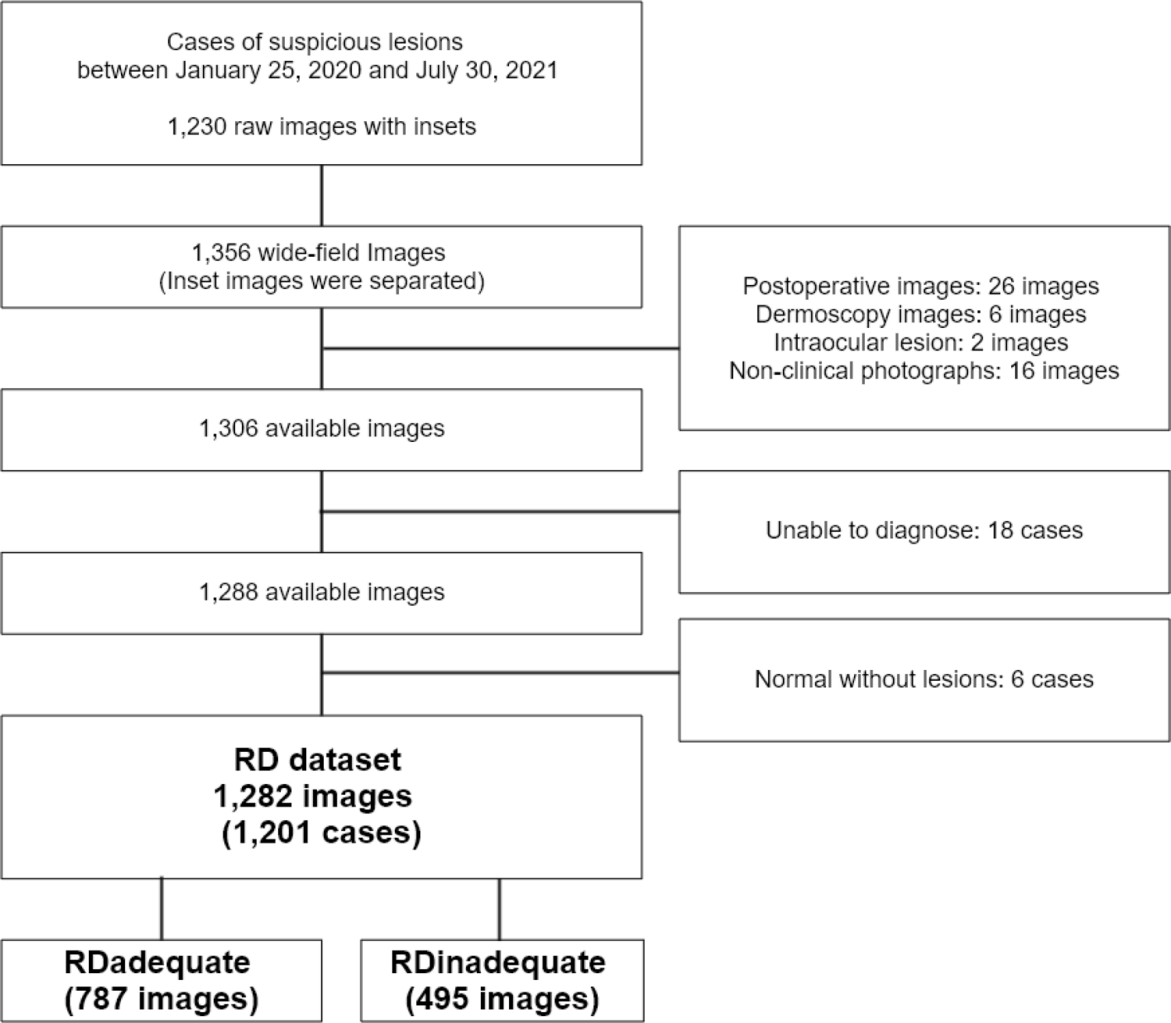
Flowchart of the RD dataset.

The RD dataset was created using images posted by a melanoma community on the Internet (https://reddit.com/r/melanoma). In the Internet community, laypersons ask about their suspicious lesions and get feedback on them. A total of 1,356 consecutive images were included by using a python library (https://github.com/aliparlakci/bulk-downloader-for-reddit) from January 25, 2020, to July 30, 2021. The following 50 images were excluded: postoperative photographs (26 images), dermoscopy images (6 images), intraocular lesion photographs (2 images), or non-clinical photographs (16 images). The ground truth was defined based on the consensus of a panel comprising four dermatologists and a plastic surgeon. Decisions were based on clinical photographs, clinical history, and if available, results of complementary testing. The ground truth was established through four rounds of votes and an agreement of ≥ 50%. If a diagnosis was not possible due to the inadequate quality of photographs (mostly for blurry image) or lack of metadata, the case was excluded (24 images). Finally, a total of 1,282 images (1,201 cases) were analyzed (Table 1, Table S1, Fig. 3). We divided the data into two subsets based on image quality assessed by the algorithm’s fine image selector submodule (RD_adequate_, 787 images and RD_inadequate_, 495 images; images were classified as ‘adequate’ if the submodule output was over 0.1; supplementary methods).

The SNU dataset was created using clinical photographs from Seoul National University Hospital, Inje University Hospital, and Hallym University Hospital (133 disorders, 2,201 images; the original dataset comprised 134 disorders, but lichen amyloidosis and amyloidosis were combined). Among the 2,201 images, 240 images from the SNU dataset are publicly available for external testing (https://doi.org/10.6084/m9.figshare.6454973; As disclosed on this repository, the reference standard was either biopsy or clinical impression)^17^. The TeleDerm dataset was created using 340 clinical photographs captured in consecutive patients at Pontificia Universidad Católica in Chile between 27 March and 30 April 2020 (the reference standard was mostly clinical impression)^18^. The Edinburgh dataset is commercially available at https://licensing.edinburgh-innovations.ed.ac.uk/i/software/dermofit-image-library.html.

### Algorithm

Model Dermatology (Build2021 https://modelderm.com) was developed, critiqued, and adjusted through academic discussions^15–17,19,23,31,32,35^. The disease classifier in Model Dermatology is an ensemble of ResNet variants that can predict 184 skin conditions^15,17^. In all the tests, the results were recorded as incorrect in cases involving out-of-distribution disorders for which the algorithm was not trained. The malignancy score was the sum of malignant outputs and 0.2 × premalignant outputs as previously defined^16^. Using the SNU dataset, the high sensitivity threshold for determining malignancy was defined as the threshold at which 90% sensitivity was obtained because the sensitivity of the attending dermatologists was at the level of 90%^17,23^. The high specificity threshold was defined as the threshold at which 80% sensitivity was obtained.

### Reader test

Using the RD dataset, we tested the performance of laypersons (using search engines) and general physicians. Six general physicians participated in the study (two doctors in their first year after getting a medical license, one first grade dermatology resident, and one first grade plastic surgery resident in Korea; two general physicians in Chile with one year and three years clinical experiences, respectively); each physician diagnosed all 1,282 included cases. The performance of the layperson was measured in a cluster manner with 32 participants (mean age [SD] = 30.7 [6.2]; 7 high school graduates and 25 college graduates; 23 females and 9 males) diagnosing the “divided batch” (39–41 cases) of the 1,282 cases without overlap. Readers were provided with wide-field images without metadata. Only for laypersons, the use of Internet search engines (about 10 min per question) was allowed to render a diagnosis. All participants participated in the reader test with a short-answer question, not a multiple choice type.

### Statistical analysis

The AUC value was calculated with R software (version 3.5.0; pROC package version 1.16.2) and Delong method was used for calculating statistical difference. Sensitivities, specificities, and Top accuracies were compared using the two-tailed *t*-test after the Shapiro test or chi-squared test. The 95% CI was generated for all samples using 2,000 stratified bootstrap replicates. In all analyses, *P* < 0.05 was taken to indicate statistical significance.

## Supplementary Information

Supplementary Information 1.

## Data Availability

The download links, ground truth, and raw results corresponding to the RD dataset are available at https://doi.org/10.6084/m9.figshare.15170853. Due to the cases deleted by users, the links of 860 cases were valid in July 2021. The whole RD dataset is available to researchers who meet the criteria for access to confidential data; requests can be made to the corresponding author, Seung Seog Han (whria78@gmail.com), or Cristian Navarrete-Dechent (ctnavarr@gmail.com). A subset of the SNU dataset (240 images) is publicly available at https://doi.org/10.6084/m9.figshare.6454973, and the Edinburgh dataset is commercially available at https://licensing.edinburgh-innovations.ed.ac.uk/i/software/dermofit-image-library.html. The TeleDerm dataset is private.

## Abbreviations

AI: Artificial intelligence
AUC: Area under the curve
CNN: Convolutional neural network
OOD: Out-of-distribution
ROC curve: Receiver operating characteristic curve
RCNN: Region-based convolutional neural network
