# Supplementary material for: The degradation of performance of a state-of-the-art skin image classifier when applied to patient-driven internet search
Source: Sci Rep. 2022 Sep 28;12:16260. doi: 10.1038/s41598-022-20632-7 (PMC9519737; doi:10.1038/s41598-022-20632-7)
Supplement: Supplementary file 1 — Supplementary Information 1. [file 41598_2022_20632_MOESM1_ESM.docx]

**SUPPLEMENTARY APPENDIX**

**Algorithm**

Model Dermatology consists of three submodules; a disease classifier, fine image selector, and blob detector. The disease classifier is an ensemble of ResNet variants that can predict 184 skin conditions in Build2021. The fine image selector is also an ensemble of ResNet variants that can rule out inadequate images via a comprehensive evaluation of their composition, focus, and brightness. The blob detector is a region-based convolutional neural network that can suggest the location of potential lesions for detection.

**Disease classifier**

The training history of our algorithm (Model Dermatology; https://modelderm.com) was described previously ^1-7^. First, the algorithm was trained using 12 benign and malignant nodules for classification of the most common skin neoplasms ^1^. As several benign disorders can mimic skin neoplasms, the algorithm should be a unified classifier that can predict a large number of disorders ^7^. The ASAN and Web datasets were mainly used for training the convolutional neural networks (CNN). The ASAN dataset was assembled with 120,780 clinical images acquired from 2003 to 2016 at the Department of Dermatology at Asan Medical Center. The Web dataset consisted of images obtained using a Python script (https://github.com/whria78/skinimagecrawler), and 300~1,500 images per disease were downloaded using two search engines (google.com and bing.com), and manually cropped and annotated based on the image findings. Further, as numerous trivial conditions may result in uncertainty, a large training dataset for the algorithm was created with the assistance of region-based convolutional neural networks ^4^. The algorithm was trained not only with typical lesions but also with various lesions generated with the assistance of a region-based convolutional neural network to reduce false positives. We trained our CNN models using a transfer learning method with ImageNet pre-trained models. Histogram normalization was performed as a preprocessing step before training the models.

The number of training image crops was 6,972,221 and only horizontal flip was applied. Ensemble of ResNet variants (SENet, SE-ResNeXt-101, SE-ResNeXt-50, ResNeSt-101, and ResNeSt-50) was used for the training of 184 classes. The hyper-parameters were set as follows: learning_rate=0.001, gamma=0.1, weight_decay=0.00001, mini_batch_size=32, solver=SGD, momentum=0.9, total_iteration=30 epoch, and step_iteration=10 epoch.

**Fine image selector module**

The fine image selector module was created in a previous study ^4^. To define the safe limits for analysis given the input, we created a fine image selector to assess image quality. Adequate images were defined as those with a detectable, well-focused skin lesion at the center of the image without any general objects. To create the fine image selector, we utilized region-based CNN and generated hundreds of thousands of raw blobs (= potential lesions) from the entire training dataset. Based on image findings, we manually classified the blobs as adequate blobs; inadequate blobs; normal or nonspecific lesional blobs; and general object blobs. With these 4-class training images, we fine-tuned SE-ResNeXt-50 to create the fine image selector module. The hyper-parameters were set as follows: learning_rate=0.001, gamma=0.1, weight_decay=0.00001, mini_batch_size=32, solver=SGD, momentum=0.9, total_iteration=30 epoch, and step_iteration=10 epoch.

The mean [SD] adequate output values using the 10 tumorous disorders of the Edinburgh dataset (1,300 images) and SNU dataset (453 images of the same 10 tumors) were 0.93 [0.15] and 0.91 [0.17], therefore, the threshold 0.90 of the adequate output was regarded to high-quality. Conversely, an arbitrary threshold of 0.10 was used as a cut-off for inadequate images. The mean [SD] adequate output using the RD dataset (1,282) was 0.42 [0.40].

**True negative test using the non-lesional background crops**


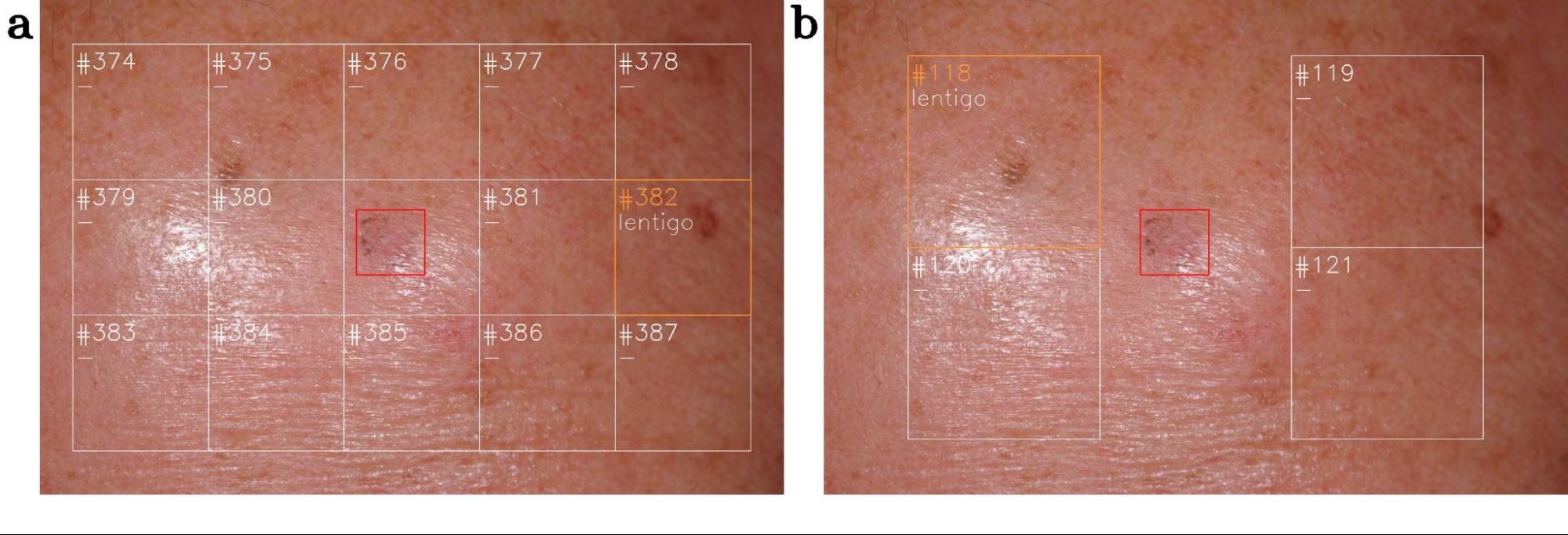


An example ISIC image (ISIC_0024289.png). The diagnostic accuracy for the non-lesional crops was evaluated assuming that the background was normal.

1. Layperson-size (5%) crop
2. Specialist-size (10%) crop

Although deep learning-based algorithms have uncertainty for untrained classes, a few studies were performed for true negatives. Here, we examined how well the algorithm could diagnose the non-lesional background crops as normal.

First, the area occupied by the lesion in the whole photograph was measured using the ISIC dataset images (<https://www.isic-archive.com>; Gallery -> 2018 JID Editorial images; 99 images; ISIC_0024262 and ISIC_0024261 are identical images and ISIC_0024262 was skipped) captured by medical professionals and the RD dataset taken by laypersons.

The lesional areas were measured as 9.4%±11.0% in the ISIC dataset and 4.8%±8.6% in the RD dataset, which implied that the lesion occupies less area in the photographs taken by a layperson. From these findings, we defined a 10% area rectangular crop as “specialist-size crop”, and 5% area rectangular crop as “layperson-size crop”.

For the non-lesional crops of the ISIC dataset, about 89.2% and 90.8% (layperson crop = 90.8%, 926/1,020 total crops; specialist crop = 89.2%, 264/296 total crops) crops were diagnosed as normal by the algorithm. For the non-lesional crops of the RD dataset, 88.0% and 87.1% (layperson-size crop = 88.0%, 13,594/15,450; specialist-size crop = 87.1%, 4,648/5,337) crops were diagnosed as normal by the algorithm. Considering the presence of some minor lesions in the background, the algorithm showed good classification performance for the normal background skin.

All ISIC images in this experiment are available at <https://doi.org/10.6084/m9.figshare.15170853>

**Analysis of diagnostic profile**

In **Figure S1A** **and S1B**, algorithms’ and physicians' 133 individual Top 1/3 accuracies were sorted independently in ascending order of accuracy. The profile of the algorithm is similar to that of two dermatologists. In **Figure S1C** and **S1D**, the accuracy of the algorithm for each diagnosis was listed in ascending order while the corresponding two dermatologist’s accuracy was drawn with red bars. Although the diagnostic performance of the algorithm was at a similar level as shown in **Figure S1A** and **Figure S1B**, the diagnostic profile of the algorithm was different from that of two dermatologists as shown in **Figure S1C** and **Figure S1D**. Different diagnostic profile was also observed for the RD dataset as shown in **Figure S2**.

**Figure S1. Distribution of multi-class classification accuracy using the SNU dataset (2,201 images)**


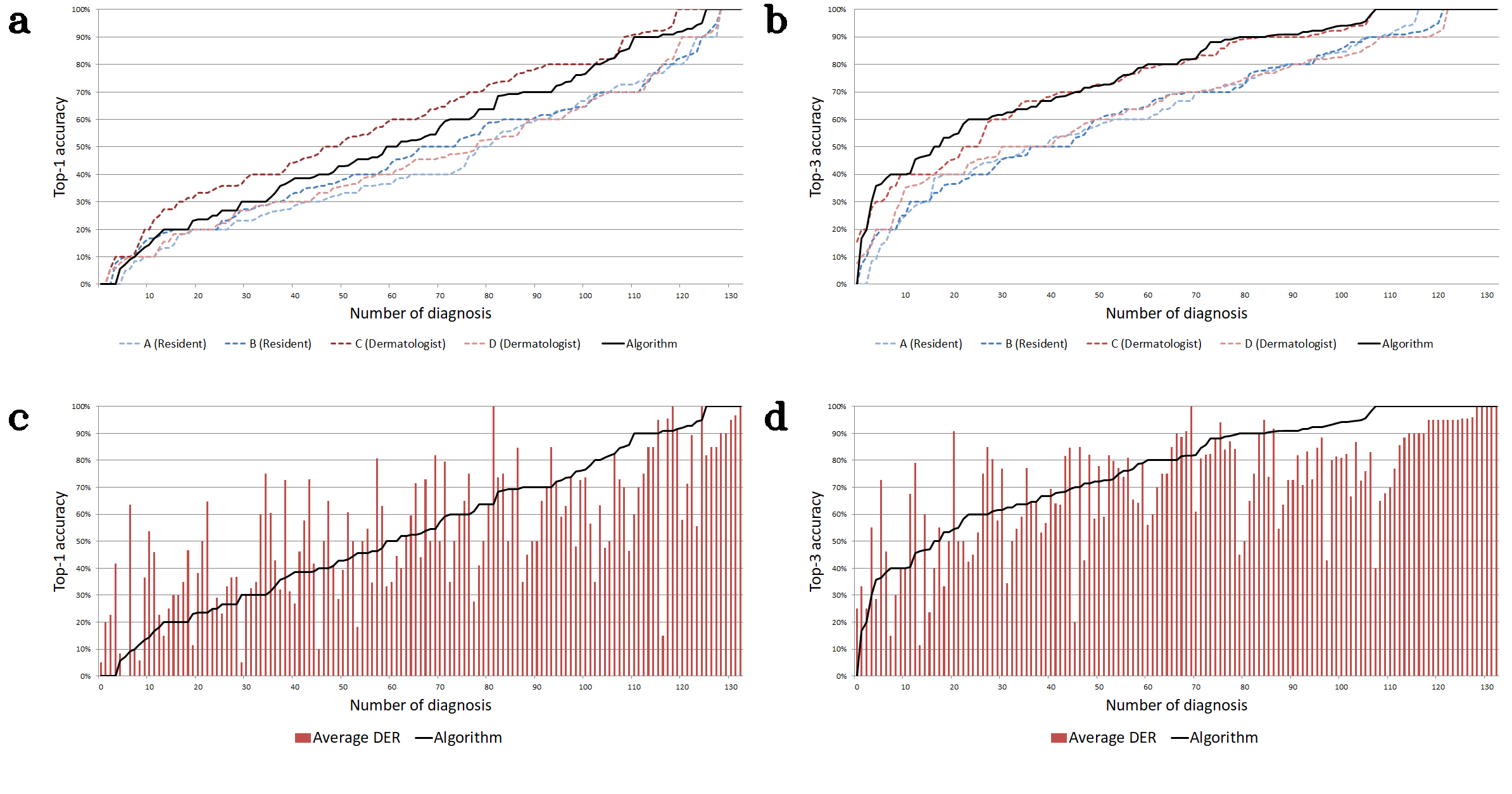


The Top-1 and Top-3 accuracy distributions were drawn using the SNU dataset (2,201 images). The results of the reader test were adapted from a previous study ^7^, and are also presented in Table S4.

**Figure S2. Distribution of multi-class classification accuracy using the RD dataset (1,282 images)**

**
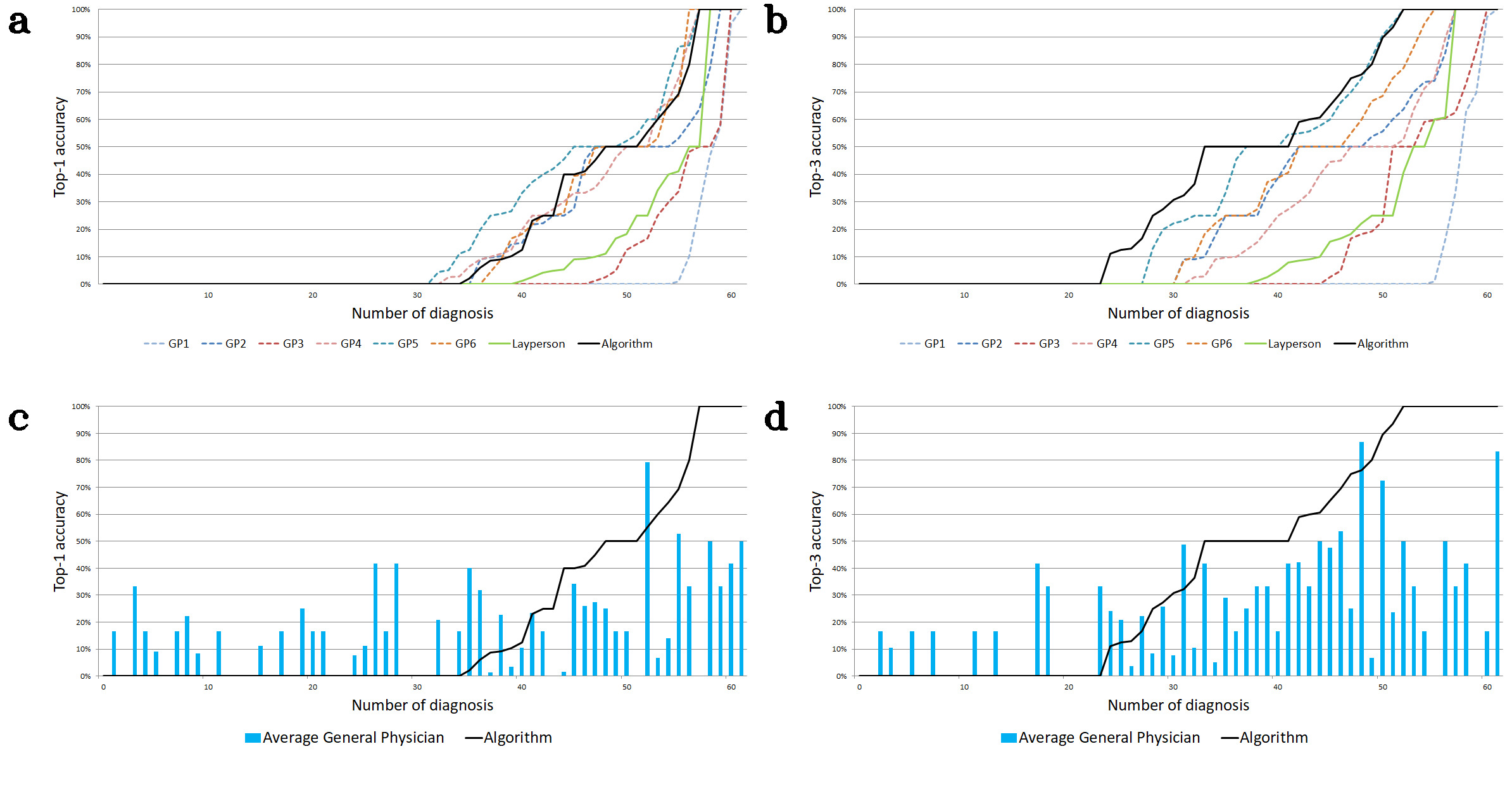
**

Layperson: the average accuracy of the layperson (cluster; 32 laypersons)

General Physician: the average accuracy of six general physicians

The Top-1 and Top-3 accuracy distributions were drawn using the RD dataset (1,282 images).

**Table S1. List of diseases of the four test datasets**

|  |  |  | **SNU** | | **Edinburgh** | | **TeleDerm** | | **RD** | |
| --- | --- | --- | --- | --- | --- | --- | --- | --- | --- | --- |
| **Category** | **Subcategory** | **Diagnosis** | **N** | **%** | **N** | **%** | **N** | **%** | **N** | **%** |
| Inflammatory | Dermatitis | Atopic dermatitis | 14 | 0.7% |  |  | 6 | 1.8% |  |  |
|  |  | Cheilitis |  |  |  |  | 1 | 0.3% |  |  |
|  |  | Chronic paronychia |  |  |  |  | 1 | 0.3% |  |  |
|  |  | Contact dermatitis | 10 | 0.5% |  |  | 28 | 8.2% | 11 | 0.9% |
|  |  | Dyshidrotic eczema | 11 | 0.5% |  |  | 2 | 0.6% |  |  |
|  |  | Erythema annulare centrifugum | 10 | 0.5% |  |  | 1 | 0.3% |  |  |
|  |  | Hand eczema | 18 | 0.9% |  |  | 10 | 2.9% |  |  |
|  |  | Lichen simplex chronicus | 17 | 0.8% |  |  | 1 | 0.3% |  |  |
|  |  | Lichenoid dermatitis* |  |  |  |  | 1 | 0.3% |  |  |
|  |  | Nummular eczema | 17 | 0.8% |  |  | 6 | 1.8% |  |  |
|  |  | Pseutotinea amiantacea |  |  |  |  | 1 | 0.3% |  |  |
|  |  | Seborrheic dermatitis | 14 | 0.7% |  |  | 10 | 2.9% |  |  |
|  |  | Unspecific eczema |  |  |  |  | 14 | 4.1% |  |  |
|  |  | Xerotic eczema | 20 | 1.0% |  |  |  |  | 1 | 0.1% |
|  | Acne/rosacea | Acne | 13 | 0.6% |  |  | 63 | 18.5% |  |  |
|  |  | Hidradenitis suppurativa |  |  |  |  | 3 | 0.9% |  |  |
|  |  | Perioral dermatitis | 19 | 0.9% |  |  | 3 | 0.9% |  |  |
|  |  | Rosacea | 19 | 0.9% |  |  | 9 | 2.6% |  |  |
|  | Autoimmune | Amicrobial pustulosis of the folds* |  |  |  |  | 1 | 0.3% |  |  |
|  |  | Bullous disease | 13 | 0.6% |  |  | 4 | 1.2% | 1 | 0.1% |
|  |  | Calcinosis cutis* |  |  |  |  | 1 | 0.3% |  |  |
|  |  | Complex aphthosis* |  |  |  |  | 1 | 0.3% |  |  |
|  |  | Cutaneous lupus |  |  |  |  | 9 | 2.6% |  |  |
|  |  | Lichen sclerosus* |  |  |  |  | 1 | 0.3% |  |  |
|  |  | Lupus erythematosus | 16 | 0.8% |  |  |  |  |  |  |
|  |  | Morphea | 20 | 1.0% |  |  | 10 | 2.9% |  |  |
|  |  | Poikiloderma (dermatomyositis) | 15 | 0.7% |  |  | 3 | 0.9% |  |  |
|  |  | Pyoderma gangrenosum | 16 | 0.8% |  |  |  |  |  |  |
|  |  | Vitiligo | 10 | 0.5% |  |  | 4 | 1.2% | 1 | 0.1% |
|  | Papulosquamous | Lichen nitidus | 10 | 0.5% |  |  | 1 | 0.3% |  |  |
|  |  | Pityriasis lichenoides chronica | 11 | 0.5% |  |  | 1 | 0.3% |  |  |
|  |  | Pityriasis lichenoides et varioliformis acuta | 21 | 1.0% |  |  |  |  |  |  |
|  |  | Pityriasis rosea | 18 | 0.9% |  |  | 2 | 0.6% |  |  |
|  |  | Psoriasis |  |  |  |  | 13 | 3.8% |  |  |
|  |  | Psoriasis (guttate) | 11 | 0.5% |  |  |  |  |  |  |
|  |  | Psoriasis (palmoplatar pustulosis) | 10 | 0.5% |  |  |  |  |  |  |
|  |  | Psoriasis (plaque) | 10 | 0.5% |  |  |  |  |  |  |
|  |  | Psoriasis (pustular psoriasis  ) | 14 | 0.7% |  |  |  |  |  |  |
|  | Others inflammatory | Acute generalized exanthematous pustulosis | 10 | 0.5% |  |  |  |  |  |  |
|  |  | Drug eruption | 21 | 1.0% |  |  |  |  |  |  |
|  |  | Erythema multiforme | 10 | 0.5% |  |  |  |  |  |  |
|  |  | Exantema* |  |  |  |  | 2 | 0.6% |  |  |
|  |  | Exfoliative dermatitis | 19 | 0.9% |  |  |  |  |  |  |
|  |  | Grover’s disease |  |  |  |  | 1 | 0.3% |  |  |
|  |  | Insect bite | 13 | 0.6% |  |  | 3 | 0.9% | 1 | 0.1% |
|  |  | Parapsoriasis |  |  |  |  |  |  | 1 | 0.1% |
|  |  | Pernio Like Eruption* |  |  |  |  | 1 | 0.3% |  |  |
|  |  | Pigmented progressive purpuric dermatosis | 10 | 0.5% |  |  |  |  | 2 | 0.2% |
|  |  | Pityriasis alba | 15 | 0.7% |  |  |  |  |  |  |
|  |  | Pityriasis amiantacea |  |  |  |  |  |  | 2 | 0.2% |
|  |  | Postinflammatory hyperpigmentation |  |  |  |  | 1 | 0.3% | 23 | 1.8% |
|  |  | Postinflammatory hypopigmentation* |  |  |  |  | 1 | 0.3% |  |  |
|  |  | Prurigo nodularis | 10 | 0.5% |  |  |  |  | 1 | 0.1% |
|  |  | Sarcoidosis* |  |  |  |  | 1 | 0.3% |  |  |
|  |  | Ulcers |  |  |  |  | 1 | 0.3% |  |  |
|  |  | Urticaria | 17 | 0.8% |  |  | 3 | 0.9% |  |  |
|  |  | Urticarial vasculitis | 18 | 0.9% |  |  |  |  |  |  |
|  |  | Vasculitis | 16 | 0.8% |  |  |  |  |  |  |
| Infectious | Viral | Condylomas | 11 | 0.5% |  |  | 1 | 0.3% |  |  |
|  |  | Eczema herpeticum | 13 | 0.6% |  |  | 1 | 0.3% |  |  |
|  |  | Fifth disease | 25 | 1.2% |  |  |  |  |  |  |
|  |  | Herpes simplex | 14 | 0.7% |  |  |  |  |  |  |
|  |  | Herpes zoster | 22 | 1.0% |  |  | 5 | 1.5% |  |  |
|  |  | Molluscum contagiosum | 14 | 0.7% |  |  | 1 | 0.3% |  |  |
|  |  | Varicella | 12 | 0.6% |  |  |  |  |  |  |
|  |  | Viral rash | 13 | 0.6% |  |  | 2 | 0.6% |  |  |
|  |  | Warts |  |  |  |  | 12 | 3.5% | 39 | 3.0% |
|  |  | Warts, flat | 10 | 0.5% |  |  |  |  |  |  |
|  |  | Warts, plantar | 10 | 0.5% |  |  |  |  |  |  |
|  | Fungal | Balanitis* |  |  |  |  | 1 | 0.3% |  |  |
|  |  | Candidal intertrigo* |  |  |  |  | 2 | 0.6% |  |  |
|  |  | Onychomycosis | 14 | 0.7% |  |  | 5 | 1.5% | 1 | 0.1% |
|  |  | Pityriasis Versicolor | 15 | 0.7% |  |  | 2 | 0.6% | 4 | 0.3% |
|  |  | Tinea |  |  |  |  | 10 | 2.9% |  |  |
|  |  | Tinea corporis | 18 | 0.9% |  |  |  |  | 1 | 0.1% |
|  |  | Tinea cruris | 15 | 0.7% |  |  |  |  |  |  |
|  |  | Tinea faciale | 13 | 0.6% |  |  |  |  |  |  |
|  |  | Tinea pedis | 10 | 0.5% |  |  |  |  | 1 | 0.1% |
|  | Bacterial | Abscess | 10 | 0.5% |  |  |  |  |  |  |
|  |  | Angular cheilitis | 11 | 0.5% |  |  | 1 | 0.3% |  |  |
|  |  | Cellulitis | 13 | 0.6% |  |  |  |  |  |  |
|  |  | Folliculitis | 17 | 0.8% |  |  | 2 | 0.6% |  |  |
|  |  | Furuncle | 10 | 0.5% |  |  | 1 | 0.3% | 1 | 0.1% |
|  |  | Impetigo | 15 | 0.7% |  |  | 1 | 0.3% |  |  |
|  |  | Inflammed cyst | 10 | 0.5% |  |  |  |  |  |  |
|  |  | Ingrowing nail | 14 | 0.7% |  |  | 2 | 0.6% |  |  |
|  |  | Paronychia | 12 | 0.6% |  |  | 1 | 0.3% | 2 | 0.2% |
|  |  | Pyoderma* |  |  |  |  | 1 | 0.3% |  |  |
|  |  | Staphylococcal scaled skin syndrome | 13 | 0.6% |  |  |  |  |  |  |
|  | Parasitic | Demodicosis* |  |  |  |  | 1 | 0.3% |  |  |
|  |  | Scabies | 15 | 0.7% |  |  |  |  |  |  |
| Neoplastic | Benign | Actinic Keratosis | 50 | 2.4% | 45 | 3.5% | 2 | 0.6% | 2 | 0.2% |
|  |  | Angiofibroma | 13 | 0.6% |  |  |  |  |  |  |
|  |  | Angiokeratoma | 10 | 0.5% |  |  |  |  | 10 | 0.8% |
|  |  | Becker nevus | 11 | 0.5% |  |  |  |  |  |  |
|  |  | Blue nevus | 10 | 0.5% |  |  |  |  | 5 | 0.4% |
|  |  | Cafe au lait macule | 11 | 0.5% |  |  |  |  |  |  |
|  |  | Cherry hemangioma |  |  |  |  |  |  | 4 | 0.3% |
|  |  | Congenital nevus | 10 | 0.5% |  |  |  |  | 4 | 0.3% |
|  |  | Cutaneous horn |  |  |  |  | 1 | 0.3% |  |  |
|  |  | Dermatofibroma | 46 | 2.2% | 65 | 5.0% | 2 | 0.6% | 31 | 2.4% |
|  |  | Dysplastic nevus |  |  |  |  |  |  | 181 | 14.1% |
|  |  | Epidermal cyst | 23 | 1.1% |  |  | 2 | 0.6% | 4 | 0.3% |
|  |  | Epidermal nevus | 11 | 0.5% |  |  |  |  | 1 | 0.1% |
|  |  | Halo nevus |  |  |  |  |  |  | 2 | 0.2% |
|  |  | Hemangioma | 37 | 1.8% | 97 | 7.5% |  |  | 20 | 1.6% |
|  |  | Inflamed nevus* |  |  |  |  | 2 | 0.6% |  |  |
|  |  | Inverted follicular keratosis* |  |  |  |  | 1 | 0.3% |  |  |
|  |  | Juvenile xanthogranuloma | 20 | 1.0% |  |  |  |  |  |  |
|  |  | Keloid | 14 | 0.7% |  |  | 2 | 0.6% |  |  |
|  |  | Lymphangioma | 10 | 0.5% |  |  |  |  |  |  |
|  |  | Melanocytic nevus | 63 | 3.0% | 331 | 25.5% | 6 | 1.8% | 490 | 38.2% |
|  |  | Melanonychia | 10 | 0.5% |  |  |  |  | 38 | 3.0% |
|  |  | Milia | 11 | 0.5% |  |  | 1 | 0.3% | 1 | 0.1% |
|  |  | Mucosal melanotic macule | 10 | 0.5% |  |  |  |  | 1 | 0.1% |
|  |  | Mucosal mucocele | 11 | 0.5% |  |  |  |  |  |  |
|  |  | Neurofibroma | 10 | 0.5% |  |  |  |  |  |  |
|  |  | Neurofibromatosis | 14 | 0.7% |  |  |  |  |  |  |
|  |  | Nevus spilus | 10 | 0.5% |  |  |  |  | 3 | 0.2% |
|  |  | Non-mucosal mucocele | 11 | 0.5% |  |  |  |  |  |  |
|  |  | Organoid nevus | 10 | 0.5% |  |  | 2 | 0.6% | 2 | 0.2% |
|  |  | Porokeratosis | 10 | 0.5% |  |  |  |  | 1 | 0.1% |
|  |  | Poroma | 10 | 0.5% |  |  |  |  |  |  |
|  |  | Pyogenic granuloma | 43 | 2.0% | 24 | 1.8% |  |  | 4 | 0.3% |
|  |  | Sebaceus hyperplasia | 19 | 0.9% |  |  |  |  |  |  |
|  |  | Seborrheic keratosis | 50 | 2.4% | 257 | 19.8% | 5 | 1.5% | 78 | 6.1% |
|  |  | Skin tag | 10 | 0.5% |  |  |  |  | 9 | 0.7% |
|  |  | Soft fibroma |  |  |  |  |  |  | 2 | 0.2% |
|  |  | Spitz nevus |  |  |  |  |  |  | 1 | 0.1% |
|  |  | Steatocystoma multiplex | 10 | 0.5% |  |  |  |  |  |  |
|  |  | Syringoma | 11 | 0.5% |  |  |  |  |  |  |
|  |  | Unilateral lentiginosis |  |  |  |  |  |  | 2 | 0.2% |
|  |  | Targetoid Hemosiderotic Hemangioma* |  |  |  |  | 1 | 0.3% |  |  |
|  |  | Telangiectatic granuloma* |  |  |  |  | 1 | 0.3% |  |  |
|  |  | Venous lake | 10 | 0.5% |  |  |  |  |  |  |
|  |  | Xanthelasma | 11 | 0.5% |  |  |  |  |  |  |
|  | Malignant | Basal cell carcinoma | 34 | 1.6% | 239 | 18.4% | 3 | 0.9% | 1 | 0.1% |
|  |  | Bowen´s disease | 37 | 1.8% | 78 | 6.0% | 1 | 0.3% | 1 | 0.1% |
|  |  | Keratoacanthoma | 18 | 0.9% |  | 0.0% |  |  | 2 | 0.2% |
|  |  | Malignant melanoma | 49 | 2.3% | 76 | 5.8% |  |  | 117 | 9.1% |
|  |  | Squamous cell carcinoma | 44 | 2.1% | 88 | 6.8% |  |  | 2 | 0.2% |
| Alopecias | Scarring | Folliculitis decalvans |  |  |  |  | 1 | 0.3% |  |  |
|  |  | Lichen planus pilaris* |  |  |  |  | 6 | 1.8% |  |  |
|  |  | Non-specified* |  |  |  |  | 1 | 0.3% |  |  |
|  | Non-scarring | Alopecia Areata | 10 | 0.5% |  |  | 1 | 0.3% |  |  |
|  |  | Androgenetic alopecia | 10 | 0.5% |  |  | 4 | 1.2% |  |  |
|  |  | Effluvium telogen* |  |  |  |  | 2 | 0.6% |  |  |
| Other |  | ABNOM | 10 | 0.5% |  |  |  |  |  |  |
|  |  | Acanthosis nigricans | 11 | 0.5% |  |  |  |  |  |  |
|  |  | Acne scar |  |  |  |  | 2 | 0.6% | 1 | 0.1% |
|  |  | Atrophic scar |  |  |  |  |  |  | 1 | 0.1% |
|  |  | Amyloidosis | 10 | 0.5% |  |  |  |  |  |  |
|  |  | Burn |  |  |  |  | 2 | 0.6% |  |  |
|  |  | Confluent reticulated papillomatosis | 13 | 0.6% |  |  |  |  |  |  |
|  |  | Crust |  |  |  |  |  |  | 11 | 0.9% |
|  |  | Dermatitis, unspecific |  |  |  |  |  |  | 2 | 0.2% |
|  |  | Erosion and laceration |  |  |  |  |  |  | 6 | 0.5% |
|  |  | Erythema ab igne | 12 | 0.6% |  |  | 1 | 0.3% |  |  |
|  |  | Erythema nodosum | 11 | 0.5% |  |  |  |  |  |  |
|  |  | Freckle | 11 | 0.5% |  |  |  |  | 2 | 0.2% |
|  |  | Galli-Galli disease* |  |  |  |  | 1 | 0.3% |  |  |
|  |  | Granuloma annulare | 10 | 0.5% |  |  |  |  | 1 | 0.1% |
|  |  | Hematoma |  |  |  |  | 2 | 0.6% | 96 | 7.5% |
|  |  | Hypertrophic Scar | 11 | 0.5% |  |  |  |  |  |  |
|  |  | Idiopathic guttate hypomelanosis | 13 | 0.6% |  |  |  |  | 1 | 0.1% |
|  |  | Keratoderma | 12 | 0.6% |  |  |  |  |  |  |
|  |  | Keratosis pilaris | 18 | 0.9% |  |  |  |  |  |  |
|  |  | Lentigo | 10 | 0.5% |  |  |  |  | 20 | 1.6% |
|  |  | Lichen amyloidosis | 15 | 0.7% |  |  |  |  |  |  |
|  |  | Lichen planus | 10 | 0.5% |  |  |  |  |  |  |
|  |  | Lichen striatus | 10 | 0.5% |  |  |  |  |  |  |
|  |  | Livedo reticularis | 10 | 0.5% |  |  |  |  |  |  |
|  |  | Melasma | 17 | 0.8% |  |  |  |  |  |  |
|  |  | Nail dystrophy | 10 | 0.5% |  |  |  |  | 5 | 0.4% |
|  |  | Nevus depigmentosus | 10 | 0.5% |  |  |  |  |  |  |
|  |  | Onycholysis | 17 | 0.8% |  |  |  |  | 1 | 0.1% |
|  |  | Ota nevus | 13 | 0.6% |  |  |  |  |  |  |
|  |  | Purpura |  |  |  |  |  |  | 8 | 0.6% |
|  |  | Portwine stain | 15 | 0.7% |  |  | 1 | 0.3% | 2 | 0.2% |
|  |  | Prurigo pigmentosa | 11 | 0.5% |  |  |  |  |  |  |
|  |  | Riehl melanosis | 11 | 0.5% |  |  |  |  |  |  |
|  |  | Scar |  |  |  |  | 3 | 0.9% | 11 | 0.9% |
|  |  | Striae distansae | 20 | 1.0% |  |  | 4 | 1.2% |  |  |
|  |  | Subungual hematoma | 17 | 0.8% |  |  |  |  |  |  |
|  |  | Telangiectasia | 15 | 0.7% |  |  |  |  | 2 | 0.2% |
|  |  | Urticaria pigmentosa | 21 | 1.0% |  |  |  |  |  |  |
| Total images |  |  | 2,101 |  | 1,300 |  | 340 |  | 1,282 |  |
| Total conditions | |  | 134 |  | 10 |  | 87 |  | 62 |  |

*: out-of-distribution disorders that were not trained by the algorithm

**Table S2. Multiclass Task – Top accuracies of the algorithms for general skin disorders in the RD Dataset**

| **RD Dataset** |  | **Algorithm** | | ***Layperson** | | **Six General Physicians** | |
| --- | --- | --- | --- | --- | --- | --- | --- |
| **1,282 images** | **Number of Images** | **Top1** | **Top-3** | **Top1** | **Top-3** | **Top1** | **Top-3** |
| Acne scar | 1 | 0.0% | 0.0% | 0.0% | 0.0% | 0.0% | 0.0% |
| Actinic keratosis | 2 | 0.0% | 50.0% | 0.0% | 0.0% | 16.7% | 41.7% |
| Angiokeratoma | 10 | 40.0% | 50.0% | 10.0% | 10.0% | 1.7% | 5.0% |
| Atrophic scar | 1 | 0.0% | 0.0% | 0.0% | 0.0% | 0.0% | 0.0% |
| Basal cell carcinoma | 1 | 0.0% | 100.0% | 0.0% | 0.0% | 33.3% | 50.0% |
| Blue nevus | 5 | 80.0% | 100.0% | 40.0% | 60.0% | 33.3% | 33.3% |
| Bullous disease | 1 | 0.0% | 0.0% | 0.0% | 0.0% | 16.7% | 16.7% |
| Cherry hemangioma | 4 | 50.0% | 50.0% | 0.0% | 25.0% | 25.0% | 29.2% |
| Congenital nevus | 4 | 50.0% | 50.0% | 25.0% | 25.0% | 16.7% | 16.7% |
| Contact dermatitis | 11 | 9.1% | 27.3% | 18.2% | 18.2% | 22.7% | 25.8% |
| Crust | 11 | 0.0% | 0.0% | 9.1% | 9.1% | 9.1% | 10.6% |
| Dermatitis unspecific | 2 | 0.0% | 0.0% | 0.0% | 0.0% | 0.0% | 0.0% |
| Dermatofibroma | 31 | 64.5% | 93.5% | 0.0% | 0.0% | 14.0% | 23.7% |
| Dysplastic nevus | 181 | 6.1% | 69.6% | 0.0% | 0.0% | 31.8% | 53.7% |
| Epidermal cyst | 4 | 25.0% | 50.0% | 25.0% | 25.0% | 16.7% | 25.0% |
| Epidermal nevus | 1 | 0.0% | 100.0% | 0.0% | 100.0% | 16.7% | 16.7% |
| Erosion & laceration | 6 | 0.0% | 16.7% | 16.7% | 16.7% | 22.2% | 22.2% |
| Freckle | 2 | 0.0% | 0.0% | 0.0% | 0.0% | 8.3% | 16.7% |
| Furuncle | 1 | 100.0% | 100.0% | 100.0% | 100.0% | 0.0% | 0.0% |
| Granuloma annulare | 1 | 0.0% | 0.0% | 0.0% | 0.0% | 0.0% | 0.0% |
| halonevus | 2 | 100.0% | 100.0% | 0.0% | 0.0% | 50.0% | 50.0% |
| Hemangioma | 20 | 45.0% | 60.0% | 5.0% | 5.0% | 27.5% | 33.3% |
| Hematoma | 96 | 2.1% | 32.3% | 9.4% | 15.6% | 40.1% | 48.8% |
| Idiopathic guttate hypomelanosis | 1 | 0.0% | 0.0% | 100.0% | 100.0% | 16.7% | 16.7% |
| Insect bite | 1 | 100.0% | 100.0% | 100.0% | 100.0% | 33.3% | 33.3% |
| Intraepithelial carcinoma | 1 | 0.0% | 0.0% | 0.0% | 0.0% | 0.0% | 0.0% |
| Keratoacanthoma | 2 | 100.0% | 100.0% | 50.0% | 50.0% | 41.7% | 41.7% |
| Lentigo | 20 | 40.0% | 65.0% | 0.0% | 0.0% | 34.2% | 47.5% |
| Malignant melanoma | 117 | 23.1% | 60.7% | 41.0% | 60.7% | 23.5% | 49.9% |
| Melanocytic nevus | 490 | 69.2% | 89.6% | 34.1% | 40.6% | 52.8% | 72.5% |
| Melanonychia | 38 | 55.3% | 76.3% | 5.3% | 7.9% | 79.4% | 86.8% |
| Milia | 1 | 0.0% | 0.0% | 0.0% | 0.0% | 0.0% | 0.0% |
| Mucosal melanotic macule | 1 | 0.0% | 0.0% | 0.0% | 0.0% | 0.0% | 0.0% |
| Nail dystrophy | 5 | 60.0% | 80.0% | 0.0% | 0.0% | 6.7% | 6.7% |
| Nevus spilus | 3 | 0.0% | 0.0% | 0.0% | 0.0% | 11.1% | 16.7% |
| Onycholysis | 1 | 0.0% | 0.0% | 0.0% | 0.0% | 0.0% | 0.0% |
| Onychomysosis | 1 | 0.0% | 0.0% | 0.0% | 0.0% | 16.7% | 16.7% |
| Orgarnoid nevus | 2 | 50.0% | 50.0% | 0.0% | 0.0% | 16.7% | 33.3% |
| Parapsoriasis | 1 | 0.0% | 0.0% | 0.0% | 0.0% | 0.0% | 0.0% |
| Paronychia | 2 | 0.0% | 50.0% | 50.0% | 50.0% | 25.0% | 33.3% |
| Pigmented progressive purpuric dermatosis | 2 | 0.0% | 50.0% | 0.0% | 0.0% | 16.7% | 16.7% |
| pityriasisamiantacea | 2 | 50.0% | 100.0% | 0.0% | 0.0% | 0.0% | 0.0% |
| Porokeratosis | 1 | 0.0% | 100.0% | 0.0% | 0.0% | 16.7% | 16.7% |
| Portwine stain | 2 | 0.0% | 0.0% | 0.0% | 0.0% | 0.0% | 0.0% |
| Postinflammatory hyperpigmentation | 23 | 8.7% | 13.0% | 4.3% | 8.7% | 1.4% | 3.6% |
| Prurigo nodularis | 1 | 0.0% | 0.0% | 0.0% | 0.0% | 0.0% | 0.0% |
| Purpura | 8 | 12.5% | 12.5% | 0.0% | 0.0% | 10.4% | 20.8% |
| Pyogenic granuloma | 4 | 25.0% | 25.0% | 0.0% | 0.0% | 0.0% | 8.3% |
| Scar | 11 | 0.0% | 36.4% | 0.0% | 0.0% | 7.6% | 10.6% |
| Seborrheic keratosis | 78 | 41.0% | 59.0% | 1.3% | 1.3% | 26.1% | 42.3% |
| Skin tag | 9 | 0.0% | 11.1% | 11.1% | 22.2% | 11.1% | 24.1% |
| Soft fibroma | 2 | 0.0% | 0.0% | 0.0% | 0.0% | 41.7% | 41.7% |
| Spitz nevus | 1 | 0.0% | 0.0% | 0.0% | 0.0% | 16.7% | 33.3% |
| Squamous cell carcinoma | 2 | 0.0% | 50.0% | 0.0% | 0.0% | 41.7% | 41.7% |
| Telangiectasia | 2 | 0.0% | 0.0% | 0.0% | 0.0% | 0.0% | 0.0% |
| Tinea corporis | 1 | 0.0% | 0.0% | 0.0% | 0.0% | 0.0% | 0.0% |
| Tinea pedis | 1 | 0.0% | 0.0% | 0.0% | 0.0% | 0.0% | 0.0% |
| Tinea versicolor | 4 | 0.0% | 75.0% | 0.0% | 0.0% | 20.8% | 25.0% |
| Unilateral lentiginosis | 2 | 0.0% | 0.0% | 0.0% | 0.0% | 0.0% | 0.0% |
| Vitiligo | 1 | 100.0% | 100.0% | 100.0% | 100.0% | 50.0% | 83.3% |
| Wart | 39 | 10.3% | 30.8% | 2.6% | 2.6% | 3.4% | 7.7% |
| Xerotic eczema | 1 | 0.0% | 0.0% | 0.0% | 0.0% | 16.7% | 33.3% |
| Accuracy |  | 39.2% | 67.2% | 19.2% | 24.4% | 36.8% | 52.9% |
| Balanced Accuracy |  | 21.2 ± 32.2% | 38.4 ± 38.6% | 12.2 ± 26.4% | 15.4 ± 29.4% | 16.8 ± 17.1% | 22.3 ± 21.5% |

* The accuracy of the layperson was calculated in a clustered way. After dividing the entire test images into 32 batches, each batch was solved by each layperson.

**Table S3. Multiclass Task – Top accuracies of the algorithms for 10 skin tumors in the Edinburgh Dataset**

| **Edinburgh** |  |  | **Algorithm** | |
| --- | --- | --- | --- | --- |
| **1300 images** |  | **Number of Images** | **TOP-1** | **TOP-3** |
|  | Actinic keratosis | 45 | 75.6% | 91.1% |
|  | Basal cell carcinoma | 239 | 67.8% | 87.0% |
|  | Intraepithelial carcinoma | 78 | 12.8% | 44.9% |
|  | Dermatofibroma | 65 | 47.7% | 64.6% |
|  | Hemangioma | 97 | 12.4% | 45.4% |
|  | Malignant melanoma | 76 | 68.4% | 89.5% |
|  | Pigmented nevus | 331 | 85.8% | 97.6% |
|  | Pyogenic granuloma | 24 | 75.0% | 91.7% |
|  | Seborrheic keratosis | 257 | 73.9% | 88.7% |
|  | Squamous cell carcinoma | 88 | 44.3% | 93.2% |
| *Accuracy* |  |  | 64.0% | 84.1% |
| *Balanced Accuracy* |  |  | 56.4 ± 26.3% | 79.4 ± 20.1% |

**Table S4. Multiclass Task – Top accuracies of the algorithms for 2,101 images of the SNU dataset**

| **SNU dataset** |  | **Algorithm** | | | **R1** | | **R2** | | **DER1** | | **DER2** | |
| --- | --- | --- | --- | --- | --- | --- | --- | --- | --- | --- | --- | --- |
| **2201 images** | **Number of Images** | **TOP-1** | **TOP-3** | **TOP-5** | **TOP-1** | **TOP-3** | **TOP-1** | **TOP-3** | **TOP-1** | **TOP-3** | **TOP-1** | **TOP-3** |
| ABNOM | 10 | 70.0% | 90.0% | 100.0% | 10.0% | 70.0% | 50.0% | 80.0% | 80.0% | 90.0% | 90.0% | 90.0% |
| Abscess | 10 | 40.0% | 70.0% | 90.0% | 20.0% | 50.0% | 10.0% | 20.0% | 10.0% | 20.0% | 10.0% | 20.0% |
| Acanthosis nigricans | 11 | 90.9% | 100.0% | 100.0% | 100.0% | 100.0% | 100.0% | 100.0% | 100.0% | 100.0% | 90.9% | 90.9% |
| Acne | 13 | 69.2% | 100.0% | 100.0% | 38.5% | 53.8% | 53.8% | 92.3% | 53.8% | 100.0% | 46.2% | 53.8% |
| Actinic keratosis | 50 | 52.0% | 82.0% | 86.0% | 56.0% | 72.0% | 48.0% | 70.0% | 44.0% | 66.0% | 36.0% | 56.0% |
| Acute generalized exanthematous pustulosis | 10 | 0.0% | 0.0% | 0.0% | 0.0% | 0.0% | 20.0% | 50.0% | 0.0% | 30.0% | 10.0% | 20.0% |
| Alopecia areata | 10 | 90.0% | 100.0% | 100.0% | 90.0% | 100.0% | 100.0% | 100.0% | 100.0% | 100.0% | 90.0% | 90.0% |
| Amyloidosis | 25 | 76.0% | 80.0% | 84.0% | 32.0% | 70.0% | 24.0% | 40.0% | 52.0% | 68.0% | 44.0% | 44.0% |
| Androgenic alopecia | 10 | 100.0% | 100.0% | 100.0% | 90.0% | 100.0% | 90.0% | 100.0% | 100.0% | 100.0% | 90.0% | 90.0% |
| Angiofibroma | 13 | 38.5% | 38.5% | 38.5% | 38.5% | 46.2% | 23.1% | 30.8% | 46.2% | 46.2% | 46.2% | 46.2% |
| Angiokeratoma | 10 | 70.0% | 100.0% | 100.0% | 60.0% | 60.0% | 30.0% | 50.0% | 60.0% | 80.0% | 40.0% | 50.0% |
| Angular cheilitis | 11 | 36.4% | 54.5% | 63.6% | 72.7% | 100.0% | 81.8% | 100.0% | 90.9% | 100.0% | 54.5% | 81.8% |
| Atopic dermatitis | 14 | 85.7% | 100.0% | 100.0% | 35.7% | 50.0% | 57.1% | 71.4% | 57.1% | 78.6% | 35.7% | 57.1% |
| Basal cell carcinoma | 34 | 73.5% | 94.1% | 100.0% | 26.5% | 47.1% | 58.8% | 79.4% | 73.5% | 88.2% | 52.9% | 73.5% |
| Becker nevus | 11 | 54.5% | 72.7% | 81.8% | 36.4% | 72.7% | 36.4% | 45.5% | 81.8% | 81.8% | 81.8% | 81.8% |
| Blue nevus | 10 | 80.0% | 100.0% | 100.0% | 80.0% | 80.0% | 60.0% | 70.0% | 50.0% | 70.0% | 20.0% | 70.0% |
| Intraepithelial carcinoma (Bowen disease) | 37 | 13.5% | 40.5% | 62.2% | 29.7% | 56.8% | 32.4% | 67.6% | 45.9% | 70.3% | 27.0% | 64.9% |
| Bullous disease | 13 | 69.2% | 92.3% | 92.3% | 76.9% | 84.6% | 61.5% | 69.2% | 61.5% | 61.5% | 76.9% | 84.6% |
| Cafe au lait macule | 11 | 100.0% | 100.0% | 100.0% | 72.7% | 90.9% | 100.0% | 100.0% | 81.8% | 90.9% | 81.8% | 100.0% |
| Cellulitis | 13 | 23.1% | 46.2% | 69.2% | 23.1% | 23.1% | 7.7% | 15.4% | 15.4% | 15.4% | 7.7% | 7.7% |
| Condyloma | 11 | 63.6% | 90.9% | 100.0% | 63.6% | 63.6% | 45.5% | 90.9% | 36.4% | 63.6% | 45.5% | 45.5% |
| Confluent reticulated papillomatosis | 13 | 84.6% | 100.0% | 100.0% | 23.1% | 84.6% | 23.1% | 46.2% | 92.3% | 100.0% | 53.8% | 76.9% |
| Congenital nevus | 10 | 70.0% | 90.0% | 100.0% | 50.0% | 60.0% | 60.0% | 70.0% | 60.0% | 70.0% | 30.0% | 30.0% |
| Contact dermatitis | 10 | 50.0% | 80.0% | 90.0% | 30.0% | 60.0% | 20.0% | 50.0% | 40.0% | 50.0% | 30.0% | 70.0% |
| Dermatofibroma | 46 | 76.1% | 89.1% | 91.3% | 63.0% | 76.1% | 69.6% | 84.8% | 82.6% | 91.3% | 63.0% | 82.6% |
| Drug eruption(Viral exanthem) | 21 | 81.0% | 90.5% | 100.0% | 23.8% | 61.9% | 19.0% | 61.9% | 47.6% | 85.7% | 47.6% | 61.9% |
| Eczema herpeticum | 13 | 38.5% | 92.3% | 92.3% | 76.9% | 76.9% | 38.5% | 46.2% | 76.9% | 92.3% | 38.5% | 76.9% |
| Epidermal cyst | 23 | 78.3% | 95.7% | 95.7% | 73.9% | 87.0% | 82.6% | 91.3% | 60.9% | 73.9% | 52.2% | 78.3% |
| Epidermal nevus | 11 | 63.6% | 90.9% | 100.0% | 72.7% | 90.9% | 63.6% | 90.9% | 54.5% | 81.8% | 45.5% | 63.6% |
| Erythema ab igne | 12 | 91.7% | 100.0% | 100.0% | 25.0% | 75.0% | 83.3% | 91.7% | 91.7% | 100.0% | 91.7% | 91.7% |
| Erythema annulare centrifugum | 10 | 20.0% | 40.0% | 40.0% | 20.0% | 30.0% | 20.0% | 30.0% | 20.0% | 40.0% | 10.0% | 40.0% |
| Erythema multiforme | 10 | 70.0% | 90.0% | 90.0% | 40.0% | 40.0% | 40.0% | 50.0% | 40.0% | 40.0% | 30.0% | 50.0% |
| Erythema nodosum | 11 | 81.8% | 90.9% | 90.9% | 36.4% | 72.7% | 72.7% | 100.0% | 54.5% | 72.7% | 45.5% | 72.7% |
| Exfoliative dermatitis | 19 | 68.4% | 89.5% | 94.7% | 73.7% | 94.7% | 73.7% | 89.5% | 100.0% | 100.0% | 47.4% | 68.4% |
| Fifth disease | 25 | 52.0% | 68.0% | 80.0% | 36.0% | 44.0% | 28.0% | 36.0% | 80.0% | 92.0% | 24.0% | 36.0% |
| Folliculitis | 17 | 23.5% | 64.7% | 76.5% | 58.8% | 58.8% | 64.7% | 88.2% | 64.7% | 76.5% | 35.3% | 52.9% |
| Freckle | 11 | 45.5% | 81.8% | 90.9% | 81.8% | 100.0% | 9.1% | 36.4% | 81.8% | 100.0% | 18.2% | 81.8% |
| Furuncle | 10 | 10.0% | 20.0% | 30.0% | 20.0% | 50.0% | 0.0% | 20.0% | 10.0% | 30.0% | 10.0% | 20.0% |
| Granuloma annulare | 10 | 60.0% | 80.0% | 100.0% | 40.0% | 80.0% | 40.0% | 70.0% | 90.0% | 90.0% | 40.0% | 50.0% |
| Guttate psoriasis | 11 | 72.7% | 90.9% | 100.0% | 27.3% | 45.5% | 27.3% | 72.7% | 72.7% | 100.0% | 45.5% | 63.6% |
| Hand eczema | 18 | 38.9% | 61.1% | 72.2% | 55.6% | 88.9% | 50.0% | 83.3% | 44.4% | 83.3% | 38.9% | 77.8% |
| Hemangioma | 37 | 43.2% | 73.0% | 83.8% | 32.4% | 54.1% | 35.1% | 67.6% | 73.0% | 89.2% | 48.6% | 70.3% |
| Herpes simplex | 14 | 42.9% | 71.4% | 78.6% | 35.7% | 71.4% | 64.3% | 71.4% | 35.7% | 50.0% | 21.4% | 35.7% |
| Herpes zoster | 22 | 63.6% | 68.2% | 68.2% | 54.5% | 59.1% | 59.1% | 63.6% | 68.2% | 68.2% | 59.1% | 59.1% |
| Hypertrophic scar | 11 | 0.0% | 36.4% | 45.5% | 27.3% | 63.6% | 63.6% | 63.6% | 27.3% | 72.7% | 18.2% | 72.7% |
| Idiopathic guttate hypomelanosis | 13 | 69.2% | 92.3% | 100.0% | 69.2% | 92.3% | 84.6% | 84.6% | 100.0% | 100.0% | 69.2% | 76.9% |
| Impetigo | 15 | 26.7% | 66.7% | 66.7% | 33.3% | 60.0% | 20.0% | 46.7% | 46.7% | 66.7% | 26.7% | 46.7% |
| Inflammed cyst | 10 | 0.0% | 30.0% | 70.0% | 20.0% | 40.0% | 20.0% | 40.0% | 30.0% | 60.0% | 10.0% | 50.0% |
| Ingrowing nail | 14 | 92.9% | 100.0% | 100.0% | 57.1% | 71.4% | 50.0% | 85.7% | 85.7% | 100.0% | 57.1% | 71.4% |
| Insect bite | 13 | 46.2% | 61.5% | 69.2% | 30.8% | 61.5% | 30.8% | 38.5% | 53.8% | 69.2% | 15.4% | 46.2% |
| Juvenile xanthogranuloma | 20 | 85.0% | 95.0% | 100.0% | 80.0% | 100.0% | 60.0% | 70.0% | 80.0% | 80.0% | 60.0% | 65.0% |
| Keloid | 14 | 42.9% | 78.6% | 92.9% | 21.4% | 64.3% | 28.6% | 64.3% | 35.7% | 78.6% | 42.9% | 50.0% |
| Keratoacanthoma | 18 | 61.1% | 72.2% | 77.8% | 61.1% | 77.8% | 61.1% | 77.8% | 33.3% | 88.9% | 22.2% | 66.7% |
| Keratoderma | 12 | 25.0% | 58.3% | 58.3% | 41.7% | 66.7% | 33.3% | 41.7% | 25.0% | 41.7% | 33.3% | 58.3% |
| Keratosis pilaris | 18 | 44.4% | 66.7% | 72.2% | 33.3% | 38.9% | 44.4% | 83.3% | 77.8% | 94.4% | 22.2% | 44.4% |
| Lentigo | 10 | 100.0% | 100.0% | 100.0% | 40.0% | 90.0% | 70.0% | 100.0% | 80.0% | 90.0% | 100.0% | 100.0% |
| Lichen nitidus | 10 | 90.0% | 90.0% | 90.0% | 80.0% | 90.0% | 70.0% | 90.0% | 100.0% | 100.0% | 70.0% | 90.0% |
| Lichen planus | 10 | 20.0% | 40.0% | 40.0% | 20.0% | 70.0% | 10.0% | 30.0% | 40.0% | 40.0% | 10.0% | 40.0% |
| Lichen simplex chronicus | 17 | 11.8% | 47.1% | 70.6% | 29.4% | 29.4% | 17.6% | 17.6% | 5.9% | 35.3% | 5.9% | 11.8% |
| Lichen striatus | 10 | 90.0% | 100.0% | 100.0% | 70.0% | 100.0% | 80.0% | 90.0% | 60.0% | 90.0% | 60.0% | 90.0% |
| Livedo reticularis | 10 | 20.0% | 50.0% | 50.0% | 20.0% | 60.0% | 70.0% | 70.0% | 30.0% | 40.0% | 30.0% | 40.0% |
| Lupus erythematosus | 16 | 25.0% | 62.5% | 75.0% | 12.5% | 25.0% | 18.8% | 25.0% | 31.3% | 31.3% | 18.8% | 37.5% |
| Lymphangioma | 10 | 30.0% | 40.0% | 40.0% | 30.0% | 30.0% | 20.0% | 20.0% | 40.0% | 40.0% | 20.0% | 20.0% |
| Malignant melanoma | 49 | 59.2% | 81.6% | 87.8% | 71.4% | 83.7% | 83.7% | 91.8% | 91.8% | 93.9% | 67.3% | 83.7% |
| Melanocytic nevus | 63 | 52.4% | 88.9% | 93.7% | 25.4% | 44.4% | 34.9% | 69.8% | 55.6% | 85.7% | 63.5% | 82.5% |
| Melanonychia | 10 | 100.0% | 100.0% | 100.0% | 90.0% | 100.0% | 100.0% | 100.0% | 80.0% | 100.0% | 90.0% | 90.0% |
| Melasma | 17 | 52.9% | 88.2% | 100.0% | 58.8% | 94.1% | 41.2% | 64.7% | 58.8% | 94.1% | 29.4% | 82.4% |
| Milia | 11 | 63.6% | 81.8% | 90.9% | 72.7% | 72.7% | 54.5% | 63.6% | 100.0% | 100.0% | 100.0% | 100.0% |
| Molluscum contagiosum | 14 | 35.7% | 92.9% | 92.9% | 35.7% | 42.9% | 35.7% | 50.0% | 35.7% | 35.7% | 28.6% | 50.0% |
| Morphea | 20 | 30.0% | 60.0% | 60.0% | 10.0% | 20.0% | 10.0% | 10.0% | 45.0% | 50.0% | 20.0% | 35.0% |
| Mucocele | 11 | 0.0% | 45.5% | 54.5% | 8.3% | 8.3% | 25.0% | 25.0% | 41.7% | 83.3% | 41.7% | 75.0% |
| Mucosal melanotic macule | 10 | 100.0% | 100.0% | 100.0% | 100.0% | 100.0% | 100.0% | 100.0% | 100.0% | 100.0% | 80.0% | 90.0% |
| Mucous cyst | 11 | 90.9% | 100.0% | 100.0% | 0.0% | 0.0% | 0.0% | 0.0% | 10.0% | 40.0% | 20.0% | 40.0% |
| Nail dystrophy | 10 | 60.0% | 100.0% | 100.0% | 10.0% | 100.0% | 60.0% | 90.0% | 80.0% | 80.0% | 70.0% | 100.0% |
| Neurofibroma | 10 | 60.0% | 90.0% | 90.0% | 0.0% | 0.0% | 20.0% | 30.0% | 60.0% | 90.0% | 60.0% | 60.0% |
| Neurofibromatosis | 14 | 14.3% | 71.4% | 85.7% | 42.9% | 57.1% | 35.7% | 57.1% | 78.6% | 92.9% | 28.6% | 71.4% |
| Nevus depigmentosus | 10 | 40.0% | 80.0% | 90.0% | 20.0% | 100.0% | 30.0% | 90.0% | 70.0% | 100.0% | 30.0% | 80.0% |
| Nevus spilus | 10 | 40.0% | 60.0% | 70.0% | 40.0% | 60.0% | 50.0% | 50.0% | 60.0% | 70.0% | 70.0% | 80.0% |
| Nummular eczema | 17 | 23.5% | 64.7% | 94.1% | 17.6% | 52.9% | 29.4% | 47.1% | 23.5% | 58.8% | 52.9% | 70.6% |
| Onycholysis | 17 | 76.5% | 88.2% | 94.1% | 76.5% | 94.1% | 76.5% | 88.2% | 70.6% | 88.2% | 76.5% | 100.0% |
| Onychomysosis | 14 | 92.9% | 100.0% | 100.0% | 85.7% | 100.0% | 92.9% | 100.0% | 92.9% | 100.0% | 85.7% | 100.0% |
| Orgarnoid nevus | 10 | 90.0% | 100.0% | 100.0% | 20.0% | 50.0% | 40.0% | 50.0% | 80.0% | 90.0% | 70.0% | 90.0% |
| Ota nevus | 13 | 53.8% | 84.6% | 84.6% | 23.1% | 38.5% | 46.2% | 69.2% | 92.3% | 92.3% | 53.8% | 69.2% |
| Palmoplantar pustulosis | 10 | 70.0% | 80.0% | 100.0% | 60.0% | 80.0% | 50.0% | 70.0% | 70.0% | 100.0% | 30.0% | 70.0% |
| Paronychia | 12 | 16.7% | 91.7% | 91.7% | 33.3% | 66.7% | 41.7% | 66.7% | 50.0% | 83.3% | 41.7% | 83.3% |
| Perioral dermatitis | 19 | 31.6% | 68.4% | 89.5% | 26.3% | 84.2% | 63.2% | 78.9% | 73.7% | 89.5% | 47.4% | 73.7% |
| Pigmented progressive purpuric dermatosis | 10 | 70.0% | 80.0% | 90.0% | 50.0% | 60.0% | 70.0% | 80.0% | 80.0% | 80.0% | 60.0% | 70.0% |
| Pityriasis alba | 15 | 80.0% | 93.3% | 93.3% | 66.7% | 86.7% | 60.0% | 60.0% | 66.7% | 73.3% | 60.0% | 86.7% |
| Pityriasis lichenoides chronica | 11 | 18.2% | 63.6% | 81.8% | 0.0% | 9.1% | 27.3% | 36.4% | 27.3% | 54.5% | 18.2% | 63.6% |
| Pityriasis lichenoides et varioliformis acuta | 21 | 33.3% | 76.2% | 95.2% | 28.6% | 47.6% | 28.6% | 38.1% | 57.1% | 71.4% | 28.6% | 76.2% |
| Pityriasis rosea | 18 | 94.4% | 94.4% | 94.4% | 33.3% | 50.0% | 33.3% | 61.1% | 77.8% | 83.3% | 33.3% | 50.0% |
| Poikiloderma | 15 | 26.7% | 53.3% | 60.0% | 66.7% | 66.7% | 40.0% | 46.7% | 53.3% | 60.0% | 20.0% | 40.0% |
| Pompholyx | 11 | 45.5% | 90.9% | 100.0% | 18.2% | 54.5% | 27.3% | 54.5% | 27.3% | 45.5% | 9.1% | 81.8% |
| Porokeratosis | 10 | 100.0% | 100.0% | 100.0% | 70.0% | 80.0% | 40.0% | 70.0% | 80.0% | 90.0% | 90.0% | 90.0% |
| Poroma | 10 | 30.0% | 40.0% | 40.0% | 30.0% | 40.0% | 20.0% | 30.0% | 10.0% | 20.0% | 0.0% | 10.0% |
| Portwine stain | 15 | 100.0% | 100.0% | 100.0% | 40.0% | 40.0% | 66.7% | 86.7% | 100.0% | 100.0% | 93.3% | 100.0% |
| Prurigo nodularis | 10 | 70.0% | 80.0% | 90.0% | 40.0% | 90.0% | 70.0% | 80.0% | 70.0% | 90.0% | 60.0% | 60.0% |
| Prurigo pigmentosa | 11 | 54.5% | 63.6% | 90.9% | 18.2% | 54.5% | 36.4% | 36.4% | 63.6% | 72.7% | 36.4% | 36.4% |
| Psoriasis | 10 | 60.0% | 90.0% | 90.0% | 50.0% | 60.0% | 60.0% | 70.0% | 60.0% | 70.0% | 40.0% | 60.0% |
| Pustular psoriasis | 14 | 7.1% | 35.7% | 57.1% | 14.3% | 28.6% | 0.0% | 7.1% | 0.0% | 42.9% | 0.0% | 14.3% |
| Pyoderma gangrenosum | 16 | 37.5% | 62.5% | 62.5% | 31.3% | 56.3% | 37.5% | 43.8% | 31.3% | 50.0% | 31.3% | 50.0% |
| Pyogenic granuloma | 43 | 72.1% | 90.7% | 95.3% | 65.1% | 79.1% | 69.8% | 93.0% | 76.7% | 97.7% | 67.4% | 86.0% |
| Riehl melanosis | 11 | 9.1% | 63.6% | 81.8% | 63.6% | 72.7% | 54.5% | 90.9% | 90.9% | 90.9% | 36.4% | 63.6% |
| Rosacea | 19 | 47.4% | 78.9% | 84.2% | 63.2% | 84.2% | 52.6% | 84.2% | 73.7% | 78.9% | 52.6% | 78.9% |
| Scabies | 15 | 26.7% | 53.3% | 66.7% | 13.3% | 20.0% | 20.0% | 20.0% | 33.3% | 40.0% | 13.3% | 26.7% |
| Sebaceus hyperplasia | 19 | 73.7% | 94.7% | 100.0% | 57.9% | 57.9% | 68.4% | 78.9% | 78.9% | 89.5% | 68.4% | 84.2% |
| Seborrheic dermatitis | 14 | 57.1% | 85.7% | 85.7% | 78.6% | 92.9% | 50.0% | 78.6% | 35.7% | 71.4% | 64.3% | 92.9% |
| Seborrheic keratosis | 50 | 92.0% | 98.0% | 98.0% | 52.0% | 82.0% | 78.0% | 94.0% | 68.0% | 90.0% | 48.0% | 76.0% |
| Skin tag | 10 | 100.0% | 100.0% | 100.0% | 90.0% | 100.0% | 80.0% | 100.0% | 100.0% | 100.0% | 100.0% | 100.0% |
| Squamous cell carcinoma | 44 | 40.9% | 75.0% | 90.9% | 40.9% | 81.8% | 50.0% | 77.3% | 34.1% | 79.5% | 47.7% | 75.0% |
| Staphylococcal scaled skin syndrome | 13 | 46.2% | 69.2% | 100.0% | 23.1% | 46.2% | 61.5% | 61.5% | 100.0% | 100.0% | 61.5% | 69.2% |
| Steatocystoma multiplex | 10 | 60.0% | 60.0% | 90.0% | 40.0% | 40.0% | 20.0% | 40.0% | 40.0% | 40.0% | 30.0% | 50.0% |
| Striae distensae | 20 | 95.0% | 100.0% | 100.0% | 100.0% | 100.0% | 95.0% | 95.0% | 100.0% | 100.0% | 100.0% | 100.0% |
| Subungal hematoma | 17 | 23.5% | 88.2% | 94.1% | 76.5% | 100.0% | 64.7% | 76.5% | 64.7% | 76.5% | 64.7% | 88.2% |
| Syringoma | 11 | 45.5% | 72.7% | 81.8% | 36.4% | 45.5% | 45.5% | 63.6% | 63.6% | 72.7% | 45.5% | 45.5% |
| Telangiectasia | 15 | 26.7% | 60.0% | 73.3% | 13.3% | 26.7% | 13.3% | 33.3% | 40.0% | 66.7% | 26.7% | 40.0% |
| Tinea corporis | 18 | 50.0% | 72.2% | 83.3% | 55.6% | 77.8% | 50.0% | 77.8% | 50.0% | 66.7% | 38.9% | 50.0% |
| Tinea cruris | 15 | 20.0% | 46.7% | 53.3% | 26.7% | 40.0% | 53.3% | 60.0% | 20.0% | 60.0% | 40.0% | 60.0% |
| Tinea faciale | 13 | 38.5% | 61.5% | 76.9% | 38.5% | 53.8% | 61.5% | 69.2% | 92.3% | 92.3% | 53.8% | 61.5% |
| Tinea pedis | 10 | 30.0% | 70.0% | 80.0% | 60.0% | 70.0% | 60.0% | 80.0% | 50.0% | 90.0% | 70.0% | 80.0% |
| Tinea versicolor | 15 | 20.0% | 66.7% | 66.7% | 46.7% | 66.7% | 46.7% | 53.3% | 66.7% | 66.7% | 26.7% | 40.0% |
| Urticaria | 17 | 82.4% | 94.1% | 94.1% | 47.1% | 82.4% | 58.8% | 88.2% | 94.1% | 94.1% | 70.6% | 70.6% |
| Urticarial vasculitis | 18 | 5.6% | 16.7% | 38.9% | 5.6% | 44.4% | 16.7% | 50.0% | 11.1% | 27.8% | 5.6% | 38.9% |
| Urticaria pigmentosa | 21 | 52.4% | 76.2% | 85.7% | 4.8% | 14.3% | 38.1% | 61.9% | 90.5% | 100.0% | 52.4% | 61.9% |
| Varicella | 12 | 50.0% | 91.7% | 91.7% | 8.3% | 41.7% | 16.7% | 33.3% | 33.3% | 75.0% | 33.3% | 66.7% |
| Vasculitis | 16 | 68.8% | 93.8% | 93.8% | 68.8% | 75.0% | 62.5% | 68.8% | 75.0% | 81.3% | 75.0% | 81.3% |
| Venous lake | 10 | 90.0% | 100.0% | 100.0% | 90.0% | 90.0% | 80.0% | 80.0% | 100.0% | 100.0% | 70.0% | 90.0% |
| Verruca plana | 10 | 20.0% | 50.0% | 80.0% | 40.0% | 80.0% | 40.0% | 50.0% | 40.0% | 60.0% | 30.0% | 50.0% |
| Viral exanthem(Drug eruption) | 13 | 38.5% | 76.9% | 76.9% | 0.0% | 15.4% | 15.4% | 53.8% | 38.5% | 76.9% | 15.4% | 53.8% |
| Vitiligo | 10 | 90.0% | 100.0% | 100.0% | 100.0% | 100.0% | 70.0% | 100.0% | 80.0% | 90.0% | 60.0% | 100.0% |
| Wart | 10 | 30.0% | 60.0% | 70.0% | 70.0% | 80.0% | 70.0% | 80.0% | 80.0% | 80.0% | 70.0% | 90.0% |
| Xanthelasma | 11 | 90.9% | 100.0% | 100.0% | 100.0% | 100.0% | 90.9% | 90.9% | 100.0% | 100.0% | 100.0% | 100.0% |
| Xerotic eczema | 20 | 30.0% | 55.0% | 70.0% | 40.0% | 55.0% | 35.0% | 40.0% | 35.0% | 45.0% | 35.0% | 55.0% |
| Accuracy |  | 54.5% | 77.6% | 84.8% | 45.1% | 64.4% | 49.1% | 66.5% | 61.1% | 77.1% | 47.2% | 66.4% |
| Balanced Accuracy |  | 54.2 ± 27.9% | 76.8 ± 21.3% | 84.0 ± 18.8% | 45.0 ± 26.2% | 64.5 ± 25.5% | 48.2 ± 24.7% | 64.5 ± 24.8% | 60.5 ± 26.6% | 75.5 ± 22.3% | 46.7 ± 25.4% | 65.4 ± 22.7% |

The algorithm analyzed multiple cropped images from the SNU dataset (133 disorders; 2,201 images; lichen amyloidosis and amyloidosis are lumped together from the original dataset of 134 disorders). Among the 2,201 images, 240 images of the SNU dataset are publicly available for external testing (<https://doi.org/10.6084/m9.figshare.6454973>).

The result of the reader test is adapted from the previous study ^7^.

**Table S5. Multiclass Task – Top accuracies of the algorithms for 240 images of the SNU dataset**

| **SNU dataset** |  | **Algorithm** | |
| --- | --- | --- | --- |
| **240 images** | **Number of Images** | **TOP-1** | **TOP-3** |
| Abscess | 2 | 0.0% | 100.0% |
| Acne | 4 | 50.0% | 100.0% |
| Actinic keratosis | 4 | 75.0% | 100.0% |
| Acute generalized exanthematous pustulosis | 1 | 0.0% | 0.0% |
| Alopecia areata | 1 | 100.0% | 100.0% |
| Amyloidosis | 3 | 100.0% | 100.0% |
| Angiofibroma | 1 | 100.0% | 100.0% |
| Angiokeratoma | 1 | 100.0% | 100.0% |
| Basal cell carcinoma | 8 | 100.0% | 100.0% |
| Becker nevus | 1 | 0.0% | 0.0% |
| Blue nevus | 1 | 100.0% | 100.0% |
| Intraepithelial carcinoma | 7 | 14.3% | 57.1% |
| Cellulitis | 4 | 50.0% | 50.0% |
| Confluent reticulated papillomatosis | 3 | 100.0% | 100.0% |
| Congenital nevus | 1 | 100.0% | 100.0% |
| Contact dermatitis | 1 | 100.0% | 100.0% |
| Dermatofibroma | 1 | 100.0% | 100.0% |
| Drug eruption(Viral exanthem) | 1 | 100.0% | 100.0% |
| Eczema herpeticum | 6 | 33.3% | 83.3% |
| Epidermal cyst | 1 | 100.0% | 100.0% |
| Epidermal nevus | 1 | 100.0% | 100.0% |
| Erythema annulare centrifugum | 1 | 100.0% | 100.0% |
| Erythema multiforme | 2 | 100.0% | 100.0% |
| Exfoliative dermatitis | 1 | 100.0% | 100.0% |
| Folliculitis | 4 | 50.0% | 50.0% |
| Furuncle | 4 | 25.0% | 25.0% |
| Granuloma annulare | 1 | 100.0% | 100.0% |
| Hand eczema | 1 | 0.0% | 0.0% |
| Hemangioma | 1 | 0.0% | 100.0% |
| Herpes simplex | 6 | 83.3% | 100.0% |
| Herpes zoster | 18 | 72.2% | 77.8% |
| Impetigo | 5 | 40.0% | 40.0% |
| Inflammed cyst | 4 | 0.0% | 25.0% |
| Insect bite | 3 | 33.3% | 33.3% |
| Juvenile xanthogranuloma | 2 | 100.0% | 100.0% |
| Keratoacanthoma | 3 | 33.3% | 66.7% |
| Lentigo | 2 | 100.0% | 100.0% |
| Lichen planus | 1 | 100.0% | 100.0% |
| Lichen simplex chronicus | 1 | 0.0% | 100.0% |
| Lupus erythematosus | 2 | 50.0% | 50.0% |
| Lymphangioma | 1 | 0.0% | 0.0% |
| Malignant melanoma | 14 | 42.9% | 78.6% |
| Melanocytic nevus | 3 | 33.3% | 100.0% |
| Morphea | 1 | 0.0% | 0.0% |
| Neurofibroma | 1 | 0.0% | 100.0% |
| Neurofibromatosis | 1 | 100.0% | 100.0% |
| Nevus spilus | 1 | 0.0% | 100.0% |
| Nummular eczema | 2 | 50.0% | 100.0% |
| Onychomysosis | 8 | 87.5% | 100.0% |
| Orgarnoid nevus | 1 | 100.0% | 100.0% |
| Palmoplantar pustulosis | 1 | 100.0% | 100.0% |
| Paronychia | 4 | 25.0% | 100.0% |
| Pompholyx | 2 | 0.0% | 50.0% |
| Prurigo pigmentosa | 4 | 50.0% | 75.0% |
| Psoriasis | 2 | 100.0% | 100.0% |
| Pustular psoriasis | 1 | 0.0% | 100.0% |
| Pyoderma gangrenosum | 1 | 100.0% | 100.0% |
| Pyogenic granuloma | 1 | 100.0% | 100.0% |
| Sebaceus hyperplasia | 2 | 50.0% | 50.0% |
| Seborrheic dermatitis | 1 | 100.0% | 100.0% |
| Seborrheic keratosis | 4 | 75.0% | 100.0% |
| Skin tag | 1 | 100.0% | 100.0% |
| Squamous cell carcinoma | 8 | 50.0% | 75.0% |
| Staphylococcal scaled skin syndrome | 2 | 50.0% | 100.0% |
| Steatocystoma multiplex | 1 | 100.0% | 100.0% |
| Subungal hematoma | 2 | 0.0% | 100.0% |
| Tinea corporis | 9 | 55.6% | 77.8% |
| Tinea cruris | 3 | 33.3% | 66.7% |
| Tinea faciale | 5 | 60.0% | 100.0% |
| Tinea pedis | 8 | 37.5% | 62.5% |
| Tinea versicolor | 7 | 28.6% | 85.7% |
| Urticaria | 1 | 100.0% | 100.0% |
| Urticaria pigmentosa | 3 | 66.7% | 66.7% |
| Varicella | 10 | 40.0% | 90.0% |
| Vasculitis | 3 | 100.0% | 100.0% |
| Verruca plana | 1 | 0.0% | 100.0% |
| Vitiligo | 1 | 100.0% | 100.0% |
| Wart | 2 | 0.0% | 50.0% |
| Xanthelasma | 1 | 0.0% | 100.0% |
| Xerotic eczema | 1 | 0.0% | 100.0% |
| *Accuracy* |  | 55.0% | 80.0% |
| *Balanced Accuracy* |  | 58.1 ± 40.4% | 82.3 ± 29.6% |

The algorithm analyzed multiple cropped images from the SNU dataset (80 disorders; 240 images). A total of 240 images of the SNU dataset are publicly available for external testing (<https://doi.org/10.6084/m9.figshare.6454973>).

**Table S6. Contingency table evaluating binary classification using the RD dataset (1,282 images)**

|  |  | **Malignancy** | |
| --- | --- | --- | --- |
| **TEST = RD (1,282 images)** |  | **Positive** | **Negative** |
| High-sensitivity threshold (TH1) | Positive | 83 | 268 |
|  | Negative | 40 | 891 |
| High-specificity threshold (TH2) | Positive | 55 | 95 |
|  | Negative | 68 | 1064 |
|  |  |  |  |
| **TEST = RDadequate (787 images)** |  | **Positive** | **Negative** |
| High-sensitivity threshold (TH1) | Positive | 63 | 189 |
|  | Negative | 22 | 513 |
| High-specificity threshold (TH2) | Positive | 44 | 66 |
|  | Negative | 41 | 636 |
|  |  |  |  |
| **TEST = RDinadequte (495 images)** |  | **Positive** | **Negative** |
| High-sensitivity threshold (TH1) | Positive | 20 | 79 |
|  | Negative | 18 | 378 |
| High-specificity threshold (TH2) | Positive | 11 | 29 |
|  | Negative | 27 | 428 |

**Table S7. Assessment of Top accuracy according to image quality using the RD dataset**

| **Test Dataset** | **Algorithm** | **Top-1** | **Top-3** |
| --- | --- | --- | --- |
| RD (1,282 images) | Algorithm | 39.2% | *67.2% |
|  | GP1 | 34.1% | 42.0% |
|  | GP2 | 37.2% | 56.7% |
|  | GP3 | 30.2% | 49.0% |
|  | GP4 | 40.7% | 43.8% |
|  | GP5 | 42.8% | 66.5% |
|  | GP6 | 35.6% | 59.2% |
|  | *mean GPs* | 36.8% | 52.9% |
| RDadequate (787 images) | Algorithm | **43.2% | **71.3% |
|  | GP1 | 33.2% | 41.9% |
|  | GP2 | 36.5% | 57.7% |
|  | GP3 | 30.5% | 49.0% |
|  | GP4 | 39.1% | 42.6% |
|  | GP5 | 39.5% | 65.4% |
|  | GP6 | 35.8% | 59.1% |
|  | *mean GPs* | 35.8% | 52.7% |
| RDinadequate (495 images) | Algorithm | 32.9% | 60.8% |
|  | GP1 | 35.6% | 42.2% |
|  | GP2 | 38.4% | 55.2% |
|  | GP3 | 29.7% | 48.7% |
|  | GP4 | 43.2% | 45.7% |
|  | GP5 | 48.1% | 68.3% |
|  | GP6 | 35.4% | 59.4% |
|  | *mean GPs* | 38.4% | 53.3% |

GP1: general physician in Korea (first year after the medical license)

GP2: 1^st^ grade PS resident in Korea

GP3: general physician in Korea (first year after the medical license)

GP4: 1^st^ grade DER resident in Korea

GP5: general physician in Chile (three year experience)

GP6: general physician in Chile (one year experience)

* = P<0.05, ** = P<0.01 (one-sample t-test)

**Table S8. Binary class Task – Sensitivity of the algorithm for each malignancy in the SNU and Edinburgh Dataset**

| **Malignant Disorders** | **Test dataset** | **No. of cases** | **Sensitivity at High Sensitivity Threshold (TH1)** | **Sensitivity at High Specificity Threshold (TH2)** |
| --- | --- | --- | --- | --- |
| Malignant melanoma | SNU | 49 | 85.7% (75.5 - 95.9) | 75.5% (63.3 - 87.8) |
|  | Edinburgh | 76 | 97.4% (93.4 - 100.0) | 89.5% (81.6 - 96.1) |
| Basal cell carcinoma | SNU | 34 | 100.0% (100.0 - 100.0) | 94.1% (85.3 - 100.0) |
|  | Edinburgh | 239 | 97.5% (95.4 - 99.2) | 90.8% (87.0 - 94.1) |
| Squamous cell carcinoma | SNU | 44 | 100.0% (100.0 - 100.0) | 95.5% (88.6 - 100.0) |
|  | Edinburgh | 88 | 100.0% (100.0 - 100.0) | 98.9% (96.6 - 100.0) |
| Intraepithelial carcinoma | SNU | 37 | 81.1% (67.6 - 91.9) | 56.8% (40.5 - 73.0) |
|  | Edinburgh | 78 | 96.2% (91.0 - 100.0) | 83.3% (74.4% - 91.0%) |

**Table S9. List of 184 conditions of the Algorithm**

| ABNOM | Congenital nevus | Hand eczema | Lymphangioma | Periungual fibroma | Senile purpura | Wart |
| --- | --- | --- | --- | --- | --- | --- |
| Abscess | Contact dermatitis | Hemangioma | Malignant melanoma | Photosensitive dermatitis | Skin tag | Xanthelasma |
| Acanthosis nigricans | Cutaneous horn | Hematoma | Melanocytic nevus | Pigmented progressive purpuric dermatosis | Soft fibroma | Xanthoma |
| Acne | Cyst | Herpes simplex | Melanonychia | Pitted keratolysis | Squamous cell carcinoma | Xerotic eczema |
| Acne scar | Depressed scar | Herpes zoster | Melasma | Pityriasis alba | Staphylococcal scalded skin syndrome |  |
| Actinic keratosis | Dermal melanosis | Herpetic whitlow | Milia | Pityriasis amiantacea | Steatocystoma multiplex |  |
| Acute generalized exanthematous pustulosis | Dermatofibroma | Hidradenitis suppurativa | Molluscum contagiosum | Pityriasis lichenoides chronica | Striae distensae |  |
| Alopecia areata | Dilated pore | Hypertrophic scar | Morphea | Pityriasis lichenoides et varioliformis acuta | Subungual hematoma |  |
| Amyloidosis | Drug eruption | Idiopathic guttate hypomelanosis | Mucocele | Pityriasis rosea | Syphilis |  |
| Androgenic alopecia | Dysplastic nevus | Impetigo | Mucosal melanotic macule | Poikiloderma | Syringoma |  |
| Anetoderma | Eccrine hidrocystoma | Infantile eczema | Mucous cyst | Pompholyx | Systemic contact dermatitis |  |
| Angioedema | Eczema herpeticum | Inflammed cyst | Nail dystrophy | Porokeratosis | Tattoo |  |
| Angiofibroma | Epidermal cyst | Ingrowing nail | Neurofibroma | Poroma | Telangiectasia |  |
| Angiokeratoma | Epidermal nevus | Insect bite | Neurofibromatosis | Portwine stain | Tinea corporis |  |
| Angular cheilitis | Erosion/Laceration | Intertrigo | Nevus depigmentosus | Postinflammatory hyperpigmentation | Tinea cruris |  |
| Atopic dermatitis | Erythema ab igne | Irritated lentigo or seborrheic keratosis | Nevus spilus | Prurigo nodularis | Tinea faciei |  |
| Basal cell carcinoma | Erythema annulare centrifugum | Irritate fibroma | Nipple eczema | Prurigo pigmentosa | Tinea pedis |  |
| Becker nevus | Erythema multiforme | Juvenile xanthogranuloma | Nonspecific (normal) | Psoriasis | Tinea versicolor |  |
| Blue nevus | Erythema nodosum | Keloid | Normal nail | Purpura | Toxic epidermal necrosis |  |
| Intraepithelial carcinoma (Bowen disease) | Exfoliative dermatitis | Keratoacanthoma | Nummular eczema | Pustular psoriasis | Ulcer |  |
| Bullous pemphigoid | Fifth disease | Keratoderma | Onycholysis | Pyoderma gangrenosum | Urticaria |  |
| Burn | Folliculitis | Keratosis pilaris | Onychomysosis | Pyogenic granuloma | Urticarial vasculitis |  |
| Cafe au lait macule | Folliculitis decalvans | Lentigo | Orgarnoid nevus | Riehl melanosis | Urticaria pigmentosa |  |
| Callus | Fordyce spot | Lichen nitidus | Ota nevus | Rosacea | Varicella |  |
| Cellulitis | Freckle | Lichen planus | Palmoplantar pustulosis | Scabies | Vascular malformation |  |
| Cheilitis | Furuncle | Lichen simplex chronicus | Panniculitis | Scar | Vasculitis |  |
| Cherry Hemangioma | Granuloma annulare | Lichen striatus | Papular urticaria | Sebaceus hyperplasia | Venous lake |  |
| Chronic eczema | Grover disease | Livedoid vasculitis | Parapsoriasis | Seborrheic dermatitis | Verruca plana |  |
| Condyloma | Guttate psoriasis | Livedo reticularis | Paronychia | Seborrheic keratosis | Viral exanthem |  |
| Confluent reticulated papillomatosis | Halo nevus | Lupus erythematosus | Perioral dermatitis | Senile gluteal dermatosis | Vitiligo |  |

**REFERENCES**

1 Han, S. S. *et al.* Classification of the clinical images for benign and malignant cutaneous tumors using a deep learning algorithm. *Journal of Investigative Dermatology* **138**, 1529-1538 (2018).

2 Han, S. S. *et al.* Interpretation of the Outputs of a Deep Learning Model Trained with a Skin Cancer Dataset. *The Journal of investigative dermatology* **138**, 2275-2277 (2018).

3 Navarrete-Dechent, C. *et al.* Automated dermatological diagnosis: hype or reality? *The Journal of investigative dermatology* **138**, 2277 (2018).

4 Han, S. S. *et al.* Keratinocytic skin cancer detection on the face using region-based convolutional neural network. *JAMA dermatology* **156**, 29-37 (2020).

5 Muñoz‐López, C. *et al.* Performance of a deep neural network in teledermatology: a single‐centre prospective diagnostic study. *Journal of the European Academy of Dermatology and Venereology* **35**, 546-553 (2021).

6 Navarrete-Dechent, C., Liopyris, K. & Marchetti, M. A. Multiclass Artificial Intelligence in Dermatology: Progress but Still Room for Improvement. *The Journal of investigative dermatology* **141**, 1325-1328 (2021).

7 Han, S. S. *et al.* Augmented intelligence dermatology: deep neural networks empower medical professionals in diagnosing skin cancer and predicting treatment options for 134 skin disorders. *Journal of Investigative Dermatology* **140**, 1753-1761 (2020).
